# An organolutetium nanosensitizer synergizes with PARP inhibition to unleash STING-mediated immunity for low-dose radioimmunotherapy

**DOI:** 10.7150/thno.124034

**Published:** 2026-01-01

**Authors:** Bingchun Zeng, Kai Ling, Qingpeng Yuan, Zeyang Chen, Guangrong Zhang, Wenyue Kang, Xuanjun Zheng, Chuanghong Liao, Youqing Mai, Zhongjie Huang, Ruibin Huang, Tiantian Zhai, Hongyan Jiang

**Affiliations:** 1 Department of Radiation Oncology, Cancer Hospital of Shantou University Medical College, Shantou 515041, China.; 2 Department of Pharmacology, Shantou University Medical College, Shantou 515041, China.; 3 Department of Radiology, The First Affiliated Hospital of Shantou University Medical College, Shantou 515041, China.; 4 Department of Radiology, Shenzhen Maternity and Child Health Care Hospital, Shenzhen 518100, China.; 5 Department of Thyroid, Breast and Hernia Surgery, General Surgery, The Second Affiliated Hospital of Shantou University Medical College, Shantou 515041, China.

**Keywords:** triple-negative breast cancer (TNBC), organolutetium nanosensitizer, low-dose radioimmunotherapy, PARP inhibition, cGAS-STING pathway

## Abstract

**Rationale:** The efficacy of radiotherapy in triple-negative breast cancer (TNBC) is often limited by an immunosuppressive tumor microenvironment (TME), requiring high radiation doses that cause systemic toxicity. There is a critical need for theranostic strategies capable of guiding therapy and amplifying the efficacy of low-dose radiation.

**Methods:** We developed a multifunctional organolutetium nanosensitizer (LSPA) for image-guided, low-dose radioimmunotherapy. Lutetium (Lu) serves as both a contrast agent for CT imaging and a radiosensitizer through the generation of reactive oxygen species (ROS). The LSPA nanoparticles were engineered to selectively accumulate in tumors and release their therapeutic payload in response to the acidic TME.

**Results:** At a low 6 Gy X-ray dose, LSPA synergized with the PARP inhibitor Olaparib to induce extensive DNA damage. This activated the cGAS-STING pathway and remodeled the TME. The treatment promoted immunogenic cell death, dendritic cell maturation, and M1 macrophage repolarization. It also decreased regulatory T cells, leading to increased CD4^+^ and CD8^+^ T cell infiltration in both primary and metastatic tumors.

**Conclusion:** This theranostic strategy suppressed primary and distant (abscopal) tumors, prevented recurrence, and established durable immune memory with low-dose irradiation. Our findings present a clinically translatable approach that combines a nanosensitizer with PARP inhibition to turn immunologically “cold” tumors into “hot” ones, thereby enhancing the efficacy of low-dose radioimmunotherapy while limiting systemic toxicity.

## Introduction

Triple-negative breast cancer (TNBC), defined by the absence of estrogen receptor, progesterone receptor, and human epidermal growth factor receptor 2 expression, is an aggressive subtype of breast cancer with limited therapeutic targets and a high risk of recurrence 1. Radiotherapy (RT) plays a crucial role in achieving local and regional tumor control in TNBC patients 2. However, its therapeutic efficacy is often limited by the requirement for high radiation doses (≥50 Gy) to induce immunogenic cell death (ICD), particularly within the inherently immunosuppressive tumor microenvironment (TME) of TNBC 3. The TME in TNBC typically exhibits hypoxia, upregulated DNA repair activity, and abundant infiltration of regulatory T cells (T_regs_), all of which collectively impair the antitumor immune responses induced by RT 4. Therefore, there is an urgent need for innovative strategies that can enhance RT efficacy at lower, safer doses 5.

Theranostic nanosensitizers, which integrate diagnostic imaging capabilities with therapeutic functionalities, present a promising approach to addressing this challenge 6-9. Nanoparticles engineered with high-atomic-number (high-Z) elements, such as Lutetium (Lu), are particularly promising candidates 10,11. Their high-Z nature facilitates strong photoelectric absorption, making them effective not only as radiosensitizers but also as contrast agents for computed tomography (CT), thus enabling image-guided therapy 12,13. In particular, Lu^3+^ has been demonstrated to generate significantly higher levels of reactive oxygen species (ROS) under X-ray irradiation compared to other lanthanides, marking it as an excellent radiosensitizing agent 14. Previous studies have shown that lanthanide-based nanoparticles can reduce required radiation doses by 40-60% 15,16, while still eliciting a robust antitumor immune response 17,18.

To further amplify the immunogenic potential of this low-dose, image-guided approach, we utilized the highly potent poly(ADP-ribose) polymerase (PARP) inhibitor, Olaparib. Olaparib is known to enhance RT efficacy, especially in homologous recombination repair-deficient TNBC, through synthetic lethality 19. By inhibiting base excision repair, Olaparib dramatically increases RT-induced DNA damage, leading to cytosolic DNA accumulation 20. This cytosolic DNA subsequently activates the cyclic GMP-AMP synthase-stimulator of interferon genes (cGAS-STING) pathway, a critical regulator of type I interferon (IFN)-mediated antitumor immunity 21,22. However, achieving sufficient synergy to robustly trigger this pathway has traditionally required high radiation doses (>30 Gy), which frequently results in significant collateral toxicity to healthy tissues 23,24.

**Figure 1:**
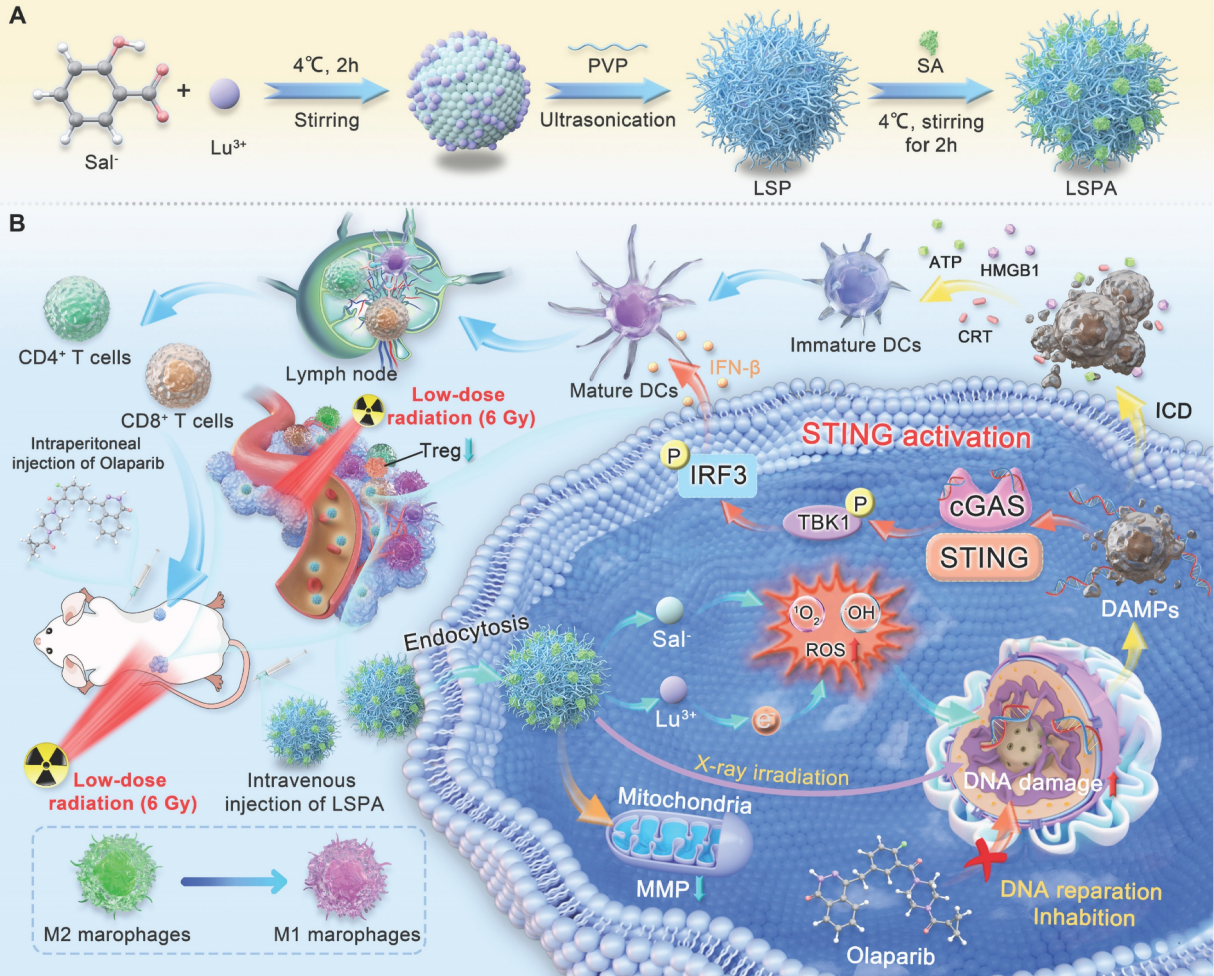
** Schematic of the LSPA-Olaparib synergistic radioimmunotherapy strategy**. **(A)** The LSPA nanoparticle is prepared via chelation of Lu^3+^ and Sal^-^, stabilized by PVP, and surface-coated with mouse serum albumin (SA). **(B)*** In vivo*, LSPA accumulates in the tumor via the EPR effect. The acidic TME triggers nanoparticle disassembly, releasing Lu^3+^ to sensitize the tumor to low-dose X-ray irradiation (RT, 6 Gy). Concurrently, Olaparib inhibits DNA repair. This synergy amplifies DNA damage, robustly activating the cGAS-STING pathway. This activation drives a systemic immune response, characterized by DC maturation, T cell infiltration, and M2-to-M1 macrophage repolarization, leading to the elimination of both primary and distant tumors.

Thus, a key challenge remains: integrating diagnostic imaging, potent radiosensitization, and synergistic PARP inhibition into a single, clinically translatable nanoplatform capable of unleashing robust immunity at a low radiation dose. To address this unmet clinical need, we developed a low-dose (<10 Gy) radioimmunotherapy strategy that combines a rationally designed, pH-responsive organolutetium theranostic nanosensitizer (LSPA) with Olaparib. As illustrated in Figure 1, LSPA nanoparticles exhibit tumor-specific accumulation, enabling CT-guided visualization. This combination of Lu-driven radiosensitization and Olaparib-mediated DNA repair inhibition dramatically enhances DNA damage and cGAS-STING activation under low dose of X-ray irradiation. The subsequent systemic immune responses reprogram the TME and eradicate both primary and abscopal tumors. To our knowledge, this is the first demonstration that coupling a theranostic nanosensitizer with a PARP inhibitor can robustly activate the STING pathway while maintaining low off-target toxicity, thus providing a practical solution to a long-standing clinical challenge.

## Results

### Design and characterization of LSPA nanoparticles

**Figure 2:**
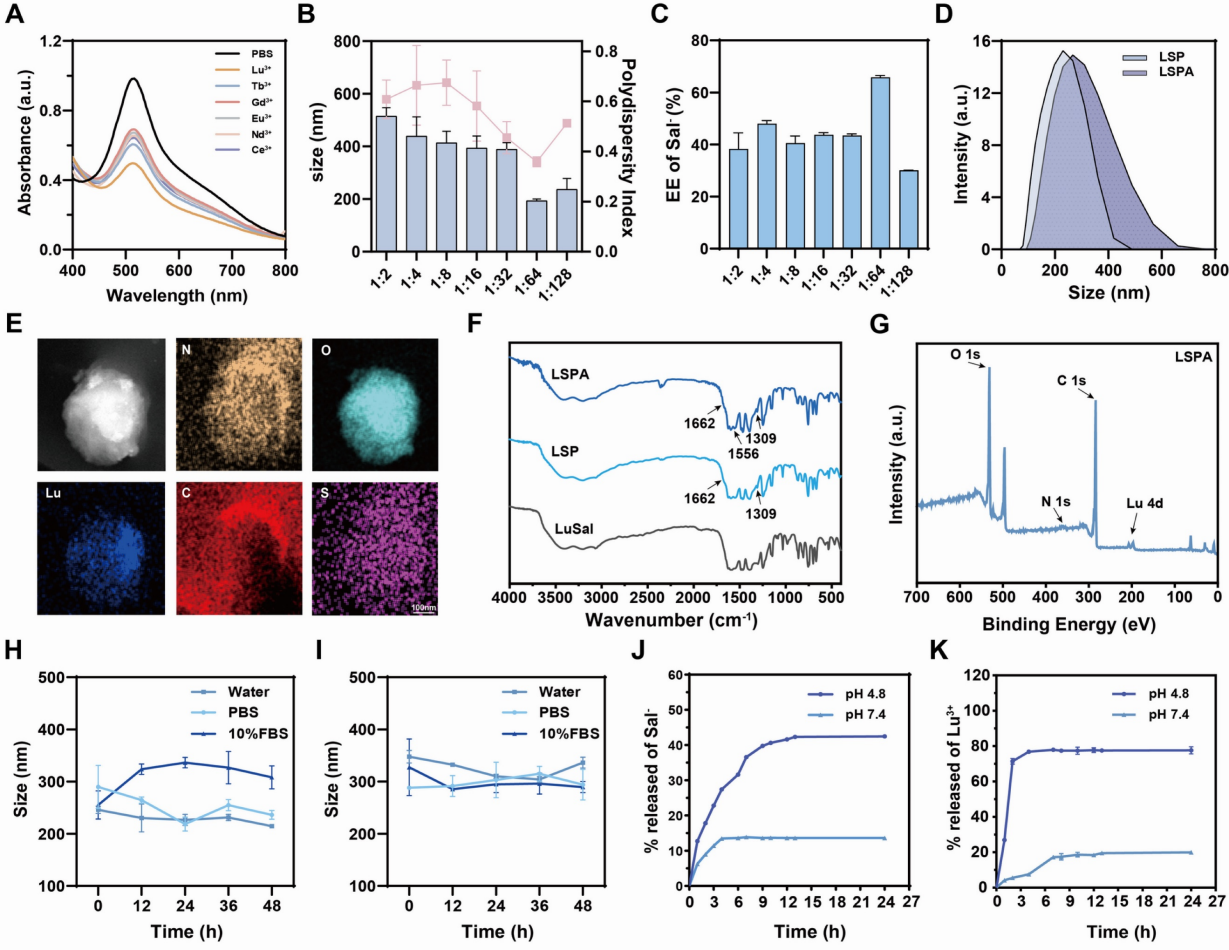
** Design and characterization of LSPA nanoparticles. (A)** UV-Vis absorption spectra of DPPH radicals incubated with lanthanide ions under 6 Gy X-ray irradiation (RT). Screening of lanthanide ions demonstrates Lu³⁺ has the highest capacity for RT-induced ROS generation. **(B)** Hydrodynamic size (nm) and polydispersity index (PDI) of LSP nanoparticles at varying Lu^3+^: Sal^-^ molar ratios (*n* = 3). **(C)** Encapsulation efficiency (EE, %) of Sal^-^ in LSP nanoparticles for different Lu^3+^: Sal^-^ molar ratios (*n* = 3). **(D)** Comparative hydrodynamic size distributions of LSP and LSPA nanoparticles. **(E)** Elemental mapping images of LSPA nanoparticles. Scale bar: 100 nm. **(F)** FT-IR spectra of LSPA, LSP, and LuSal. **(G)** XPS spectra of LSPA. **(H-I)** Hydrodynamic size changes of LSP and LSPA nanoparticles in water, PBS, and 10% FBS over 48 h (*n* = 3).** (J-K)** pH-responsive release profiles of Sal^-^ and Lu^3+^ from LSPA across various pH environments over 24 h (*n* = 3). Data are presented as mean ± SD.

High-atomic-number (high-Z) lanthanide metals enhance radiosensitization by increasing photoelectron and Auger electron production via the Compton and photoelectric effects, thereby amplifying ROS generation and tumor cell damage 17. To develop an effective theranostic agent, we first evaluated six lanthanide ions for their capacity to generate ROS under X-ray irradiation, which represents a key factor in determining radiosensitizing efficacy. Using the 2,2-diphenyl-1-picrylhydrazyl (DPPH) scavenging assay 25, we observed that Lu^3+^ elicited the greatest reduction in DPPH absorbance at 517 nm after exposure to a 6 Gy dose of X-ray irradiation, demonstrating superior performance compared to Ce^3+^, Nd^3+^, Eu^3+^, Gd^3+^, and Tb^3+^ (Figure 2A). Normalization analysis confirmed that Lu^3+^ demonstrated the highest ROS-generating capacity (Figure S1), establishing it as the optimal candidate for our nanoradiosensitizer.

Based on these findings, we prepared pH-responsive organolutetium nanoparticles (LuSal@PVP, referred to as LSP) via chelation of Lu^3+^ with salicylate (Sal^-^) ions, stabilized by polyvinylpyrrolidone (PVP). Optimization studies identified a Lu^3+^: Sal^-^ molar ratio of 1:64 as ideal, yielding nanoparticles with a hydrodynamic diameter of 195.27 ± 4.98 nm, a polydispersity index (PDI) of 0.36 ± 0.02, and a high Sal^-^ encapsulation efficiency of 65.84 ± 2.17 % (Figure 2B-C, S2). Transmission electron microscopy (TEM) revealed a spherical morphology with a core diameter of approximately 100 nm (Figure S3). To enhance biocompatibility and prolong systemic circulation, LSP nanoparticles were surface-functionalized with mouse serum albumin (SA) to form LSPA. The successful surface coating was verified by an increase in hydrodynamic diameter to 321.67 ± 6.08 nm and a corresponding shift in zeta potential (Figure 2D, S4). TEM imaging revealed a distinct SA shell surrounding the nanoparticle core, leading to an increased particle diameter of approximately 200 nm (Figure S5). Elemental mapping analysis confirmed the co-localization of Lu, C, O, N, and S, thereby validating the successful assembly of LSPA (Figure 2E).

The chemical composition was further characterized by Fourier-transform infrared (FT-IR) and X-ray photoelectron spectroscopy (XPS). The FT-IR spectrum of LSPA exhibited characteristic absorption peaks corresponding to PVP (1662 and 1309 cm^-1^) 26,27, as well as a distinct amide II band (1556 cm^-1^) originating from the SA coating (Figure 2F) 28. The XPS spectrum confirmed the presence of constituent elements (Figure 2G), and high-resolution analysis of the Lu 4d region exhibited peaks at 197.47 eV (4d_5/2_) and 206.75 eV (4d_3/2_), which are characteristic of the stable Lu^3+^ oxidation state (Figure S6) 29. Inductively coupled plasma mass spectrometry (ICP-MS) and UV-Vis spectroscopy quantified the LSPA composition as 19.8 ± 0.1 μM Lu^3+^ and 3.51 ± 0.02 mM Sal^-^ in a 600 μg mL^-1^ LSPA nanoparticle dispersion.

To improve biocompatibility and stability in biological environment, the nanoparticles were surface-functionalized with SA, a common approach in advanced nanocarriers 30,31 The SA concentration was optimized to 165 µg mL^-1^, a concentration that formed a uniform protective shell. This coating minimized nanoparticle aggregation in serum-containing media, maintained pH-responsive behavior, and maximized the payload-to-mass ratio. Colloidal stability assays demonstrated that while uncoated LSP nanoparticles aggregated in serum-containing media, the SA-coated LSPA nanoparticles remained stable in water, PBS, and 10% FBS for over 48 h, highlighting the critical role of the albumin shell in preventing aggregation (Figure 2H-I). Importantly, LSPA exhibited pH-responsive drug release behavior. In a simulated acidic endosomal/lysosomal environment (pH 4.8), 77.60 ± 2.01 % of Lu^3+^ and 42.47 ± 0.07 % of Sal^-^ were released within 24 h. In contrast, under physiological conditions (pH 7.4), minimal release was observed (19.95 ± 0.77 % for Lu^3+^ and 13.64 ± 0.04 % for Sal^-^) (Figure 2J-K). This differential release profile is attributed to the protonation of salicylate under acidic conditions, which destabilizes its coordination with Lu^3+^ and thereby facilitates targeted release at the tumor site 32. The extracellular pH of the TME typically ranges from 6.0 to 7.0 33. The selection of pH 4.8 was deliberately made to simulate the more acidic conditions present within the endo-lysosomal compartments of tumor cells, which are the expected sites of nanoparticle disassembly following cellular internalization 34. LSPA was incubated in the acid buffer (pH 4.8) to simulate this environment and then analyzed for morphology (Figure S7). TEM images show clear loss of spherical structure and significant nanoparticle disassembly under acidic conditions. This supports our proposed mechanism: salicylate protonation destabilizes the nanoparticles, triggering therapeutic payload release.

To further validate the structural integrity of LSPA nanoparticles under biologically relevant conditions, TEM was employed to examine the nanoparticles following 24 h incubation in cell culture media supplemented with 10% FBS or 10 µg mL^-1^ heparin. The TEM images demonstrated that the LSPA nanoparticles maintained their well-defined spherical morphology, with no evidence of aggregation or degradation, thereby supporting their high degree of stability (Figure S8).

### *In vitro* radiosensitization by LSPA

**Figure 3:**
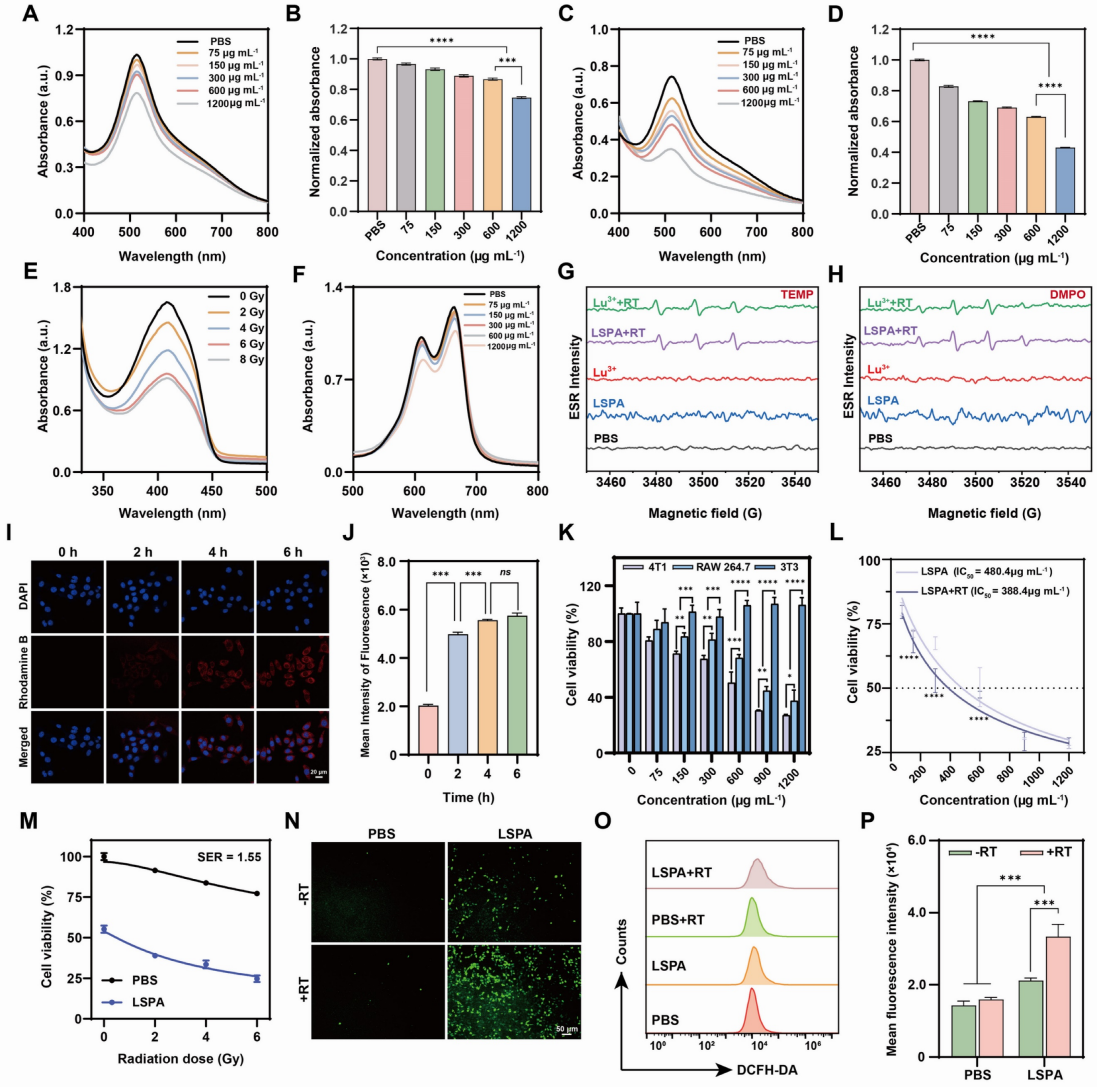
***In vitro* radiosensitization by LSPA. (A)** UV-Vis absorption spectra of DPPH radicals treated with increasing LSPA concentrations (0-1200 µg mL^-1^). **(B)** Normalized absorbance of DPPH radicals at 517 nm across LSPA concentrations (*n* = 3). **(C-D)** Corresponding UV-Vis spectra and normalized absorbance of DPPH radicals with various LSPA concentrations under 6 Gy X-ray irradiation (RT,* n* = 3). **(E)** UV-Vis spectra of DPBF in response to LSPA under varying X-ray doses. **(F)** UV-Vis spectra of MB influenced by various LSPA concentrations under RT. **(G-H)** ESR spectra of TEMP (for ^1^O_2_) and DMPO (for •OH) in the presence of Lu^3+^ or LSPA with or without RT.** (I-J)** Confocal laser scanning microscopy (CLSM) images and flow cytometry fluorescence of 4T1 cells incubated with Rhodamine B-labeled LSPA (0-6 h). Scale bar: 20 μm. **(K)** Cell viability assays of 4T1, RAW 264.7, and 3T3 cells exposed to LSPA concentrations (*n* = 3). **(L)** Viability of 4T1 cells treated with LSPA, with or without RT (*n* = 3). **(M)** Viability of 4T1 cells under various X-ray doses with LSPA (*n* = 3). **(N)** CLSM images of DCFH-DA-stained 4T1 cells treated with PBS (control) and LSPA with or without RT. Scale bar: 50 μm. **(O-P)** Flow cytometry analysis and fluorescence of DCFH-DA-stained 4T1 cells treated with PBS (control) and LSPA with or without RT (*n* = 3). Data are presented as mean ± SD; *ns*: no significance; *P < 0.05; **P < 0.01; ***P < 0.001.

We subsequently assessed the radiosensitizing capacity of LSPA. In cell-free assays, LSPA demonstrated concentration-dependent ROS generation, which was significantly amplified by 6 Gy X-ray irradiation (Figure 3A-D). This enhancement is attributed to Lu^3+^-mediated water radiolysis. Using 1,3-diphenylisobenzofuran (DPBF) and methylene blue (MB), we verified dose-dependent singlet oxygen (^1^O_2_) generation, which reached a plateau at 6 Gy (Figure 3E), as well as concentration-dependent hydroxyl radical (•OH) production (Figure 3F). Electron spin resonance (ESR) spectroscopy provided additional evidence, revealing characteristic signals for both ^1^O_2_ and •OH exclusively upon X-ray irradiation of LSPA (Figure 3G-H), thereby confirming its function as a radiosensitizer.

The therapeutic potential of LSPA was assessed in 4T1 TNBC cells. Cellular uptake studies using Rhodamine B-labeled LSPA showed rapid internalization, reaching saturation within 6 h (Figure 3I-J, S9). LSPA exhibited selective cytotoxicity, being significantly more toxic to 4T1 tumor cells (IC_50_ = 480.4 µg mL^-1^) than to normal 3T3 fibroblasts and RAW264.7 macrophages (Figure 3K). This cytotoxic effect is partially attributable to the release of Sal^-^ (Figure S10) 35, demonstrating its dual role as a structural and bioactive component.

The radiosensitizing efficacy of LSPA in 4T1 cells was potent. When combined with 6 Gy irradiation, LSPA exhibited a reduced IC_50_ of 388.4 µg mL^-1^ (Figure 3L), resulting in a sensitizer enhancement ratio (SER) of 1.55 (Figure 3M). This enhancement in cytotoxicity was mechanistically associated with a dramatic increase in intracellular ROS levels, as demonstrated by DCFH-DA staining and quantified through flow cytometry analysis (Figure 3N-P). The increase in the generation of intracellular ^1^O_2_ and •OH was also observed (Figure S11). These findings confirm that LSPA functions effectively as a nanosensitizer by amplifying the cytotoxic effects of low-dose radiation.

### Synergistic amplification of DNA damage and apoptosis

**Figure 4:**
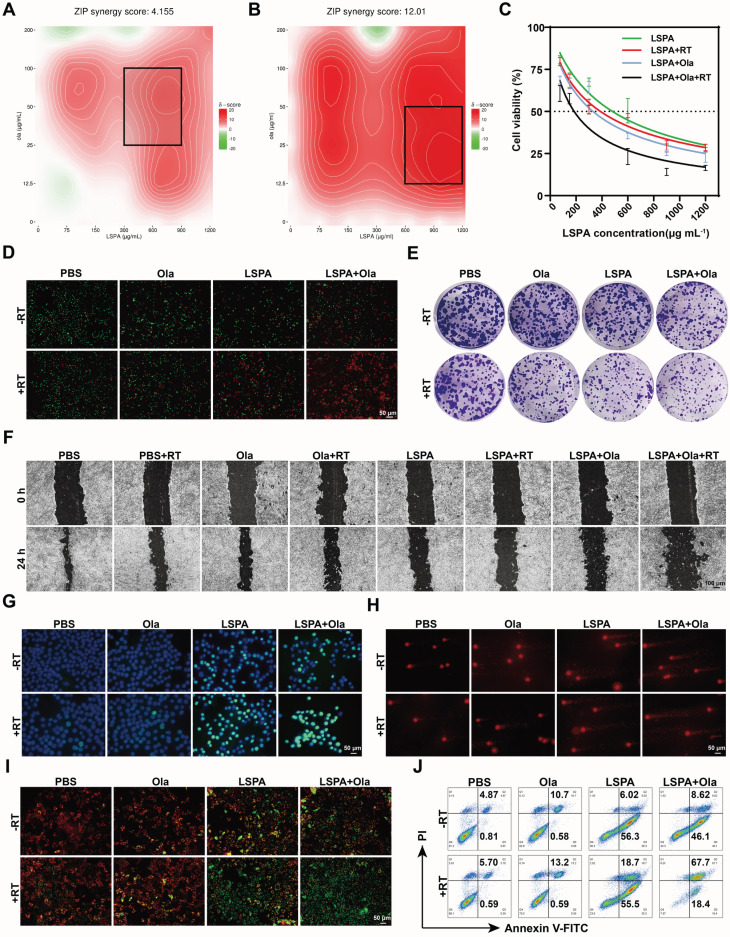
** Synergistic amplification of DNA damage and apoptosis. (A-B)** SynergyFinder analysis of 4T1 cell viability via dose-response matrices for LSPA (0-1200 μg mL^-1^) and Olaparib (Ola, 0-200 μg mL^-1^) with or without 6 Gy X-ray irradiation (RT). The black squares represent the areas exhibiting pronounced synergistic interaction between LSPA and Olaparib. **(C)** Cytotoxicity assessment of 4T1 cells treated with LSPA alone or in combination with Olaparib, with or without RT (*n* = 3). Data are presented as mean ± SD. **(D)** Live/dead staining (Calcein-AM/PI) of 4T1 cells post-treatment. Scale bar: 50 μm. **(E)** Colony formation assays showing surviving fractions after treatments (crystal violet staining). **(F)** Scratch wound healing assays quantifying 4T1 migration following the indicated treatments. Scale bar: 100 μm. **(G)** γ-H2AX immunofluorescence staining (a marker for DSBs) of 4T1 cells following the indicated treatments (DAPI counterstaining). Scale bar: 50 μm. **(H)** Comet assay images (DNA fragmentation) of 4T1 cells following the indicated treatments. Scale bar: 50 μm. **(I)** Mitochondrial depolarization (JC-1 assay) of 4T1 cells following the indicated treatments. Scale bar: 50 μm. **(J)** Apoptosis detection via Annexin V-FITC/PI flow cytometry of 4T1 cells following the indicated treatments.

Building upon the radiosensitizing properties of LSPA, we investigated its synergy with the PARP inhibitor Olaparib. A dose-response SynergyFinder matrix analysis based on the zero interaction potency (ZIP) model demonstrated a strong synergistic interaction between LSPA and Olaparib specifically under X-ray irradiation (ZIP score = 12.01), in contrast to a merely additive effect without irradiation (ZIP score = 4.155) (Figure 4A-B) 36. The combination of LSPA (600 μg mL^-1^) and Olaparib (50 μg mL^-1^) was identified as optimally synergistic and used for subsequent experiments. This combination therapy (LSPA + Olaparib + RT) dramatically lowered the LSPA IC_50_ to 185.2 µg mL^-1^, far exceeding the efficacy of LSPA + RT (IC_50_ = 388.4 µg mL^-1^) or LSPA + Olaparib (IC_50_ = 330.9 µg mL^-1^) (Figure 4C, S12).

The synergistic cytotoxicity was confirmed by live/dead staining, where the triple-combination therapy resulted in near-complete cell death (Figure 4D). This enhanced cytotoxicity corresponded to a significant suppression of long-term survival and metastatic capacity, as evidenced by colony formation and scratch wound healing assays. The LSPA + Olaparib + RT group exhibited the lowest clonogenic survival rate (3.90 ± 0.58%) and minimal wound closure (20.00 ± 7.23%) (Figure 4E-F, S13-14).

Mechanistically, we attributed this synergistic effect to an extensive accumulation of DNA damage. Immunofluorescence staining for γ-H2AX, a well-established biomarker of DNA double-strand breaks (DSBs) 37, demonstrated a significantly increased γ-H2AX fluorescence intensity (fluorescence intensity per nucleus), far exceeding that of any other treatment (Figure 4G, S15). Comet assays further supported these findings, revealing extensive DNA fragmentation characterized by significantly elongated comet tails in the triple-combination treatment group (Figure 4H, S16). This heightened genotoxic stress resulted in mitochondrial dysfunction 38, as evidenced by a near-complete loss of mitochondrial membrane potential (MMP) measured by JC-1 staining (Figure 4I). Consequently, a substantial induction of apoptosis was observed, with 67.53 ± 1.76% of 4T1 cells undergoing late-stage apoptosis in the LSPA + Olaparib + RT group (Figure 4J, S17), thereby confirming that enhanced genotoxic stress drives synergistic cell death. This demonstrates that the synergy between LSPA-radiosensitization and PARP inhibition is a powerful strategy to trigger overwhelming and irreparable DNA damage.

To confirm that the radiosensitization effect of LSPA NPs is attributable to their Lu^3+^ and Sal^-^ components, we conducted cellular assays (ROS and γ-H2AX) demonstrating that free Lu^3+^ (19.8 ± 0.1 μM) and Sal^-^ (3.51 ± 0.02 mM) ions exert a radiosensitizing effect comparable to that of LSPA nanoparticles (600 μg mL^-1^) following irradiation (Figure S18). Specifically, both groups exhibited similar increases in intracellular ROS generation and comparable levels of DNA double-strand breaks, as assessed by γ-H2AX immunofluorescence staining. These findings collectively indicate that the therapeutic mechanism is mediated by the intracellular release of Lu^3+^ and Sal^-^. Furthermore, the “Lu^3+^” and “Lu^3+^ + RT” groups were excluded from *in vivo* experiments due to insufficient tumor accumulation of free ions, which may hinder the achievement of therapeutic concentrations.

### Synergistic therapy induces ICD and STING activation

**Figure 5:**
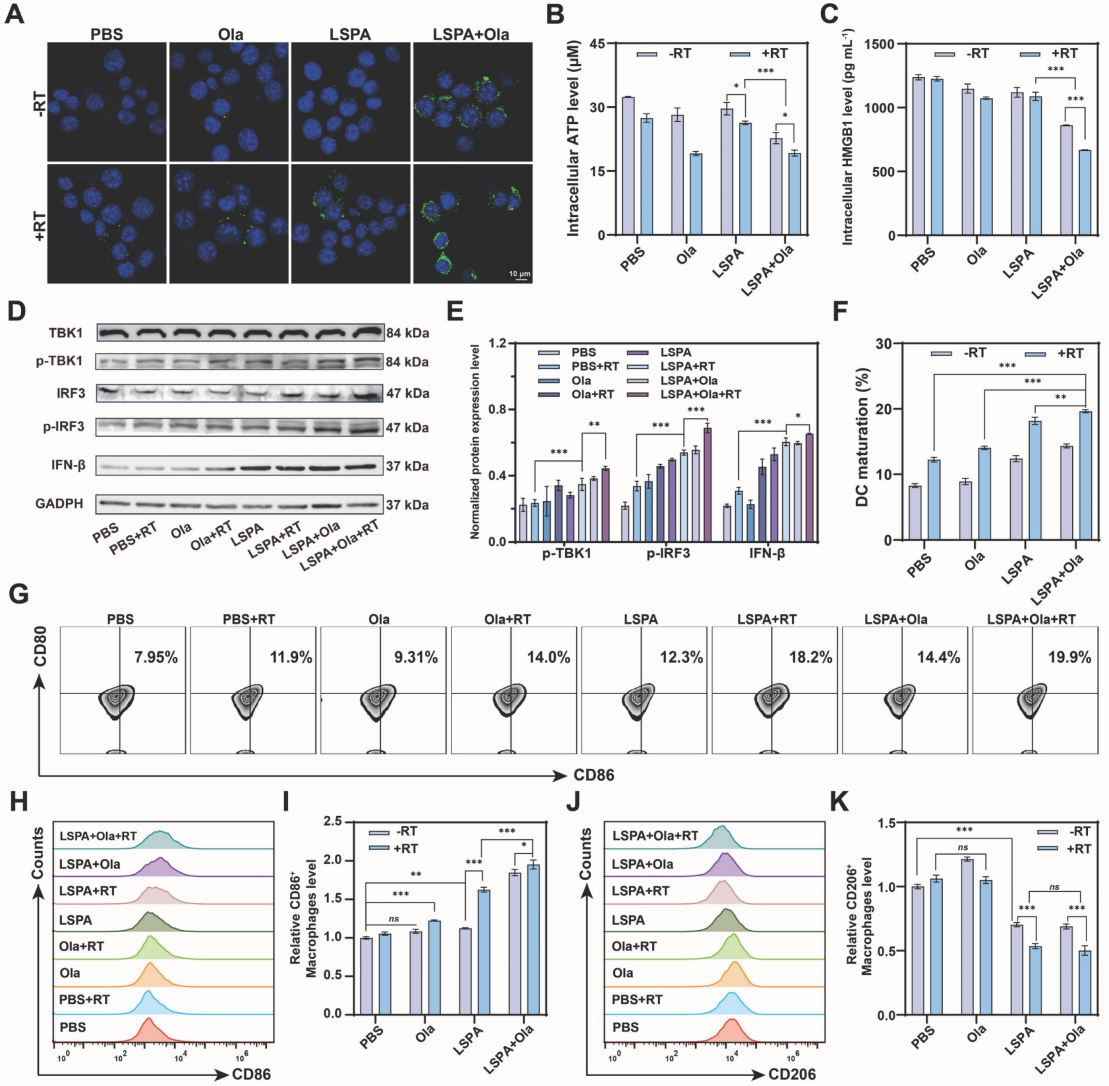
** Synergistic therapy induces ICD and STING activation. (A)** Immunofluorescence staining of CRT surface exposure in 4T1 cells post-treatment. Scale bar: 10 μm. **(B-C)** Quantification of intracellular ATP and HMGB1 levels in 4T1 cells following the indicated treatments (*n* = 3). **(D)** Western blot analysis of cGAS-STING pathway activation biomarkers (p-TBK1, p-IRF3, IFN-β) in DC2.4 cells treated with CM from 4T1 cells. **(E)** Quantification of p-TBK1, p-IRF3, and IFN-β protein levels (*n* = 3). **(F)** Percentage of mature DC2.4 cells (CD80^+^ CD86^+^, *n* = 3). **(G)** Representative flow cytometry plots of mature DCs among DC2.4 cells. **(H-I)** Flow cytometry analysis of M1-repolarized RAW264.7 macrophages (CD86⁺) and the normalized levels (*n* = 3). **(J-K)** Flow cytometry analysis of M2-polarized RAW264.7 macrophages (CD206^+^) and the normalized levels (*n* = 3). Ola: Olaparib; RT: 6 Gy X-ray irradiation. Data are presented as mean ± SD. *ns*: no significance; *P < 0.05; **P < 0.01; ***P < 0.001.

Given that extensive DNA damage serves as a potent trigger for innate immunity 39, we investigated whether our synergistic therapeutic strategy could activate the cGAS-STING pathway. The combination of LSPA + Olaparib + RT induced ICD hallmarks in 4T1 cells, including robust calreticulin (CRT) exposure on the cell surface, as well as significant extracellular release of ATP and high-mobility group box 1 (HMGB1), as indicated by reduced intracellular levels (Figure 5A-C) 40.

To evaluate downstream immune activation, we treated DC2.4 cells (immature murine dendritic cells) with conditioned medium (CM) from the treated 4T1 cells. CM obtained from the LSPA + Olaparib + RT group induced the most robust activation of the STING pathway, as evidenced by significantly enhanced phosphorylation of TBK1 and IRF3 (p-TBK1 and p-IRF3), along with increased expression of the downstream effector IFN-β (Figure 5D-E) 41. This potent STING activation corresponded to improved antigen-presenting cell function, as the LSPA + Olaparib + RT group induced the highest rate of DC maturation (19.9 % CD80^+^CD86^+^ cells) (Figure 5F-G).

Furthermore, our therapeutic strategy effectively reprogrammed the immunosuppressive phenotype of macrophages. When M2-polarized RAW264.7 macrophages were treated with CM, the LSPA + Olaparib + RT group induced the most significant repolarization towards a pro-inflammatory M1 phenotype (CD86^+^) while reducing the M2 population (CD206⁺), achieving the highest M1/M2 ratio (Figure 5H-K). These findings demonstrate that the synergy between LSPA-mediated radiosensitization and PARP inhibition transforms dying tumor cells into a powerful *in situ* vaccine, thereby robustly activating STING-dependent antitumor immunity.

### LSPA enables *in vivo* tumor targeting and theranostics

**Figure 6:**
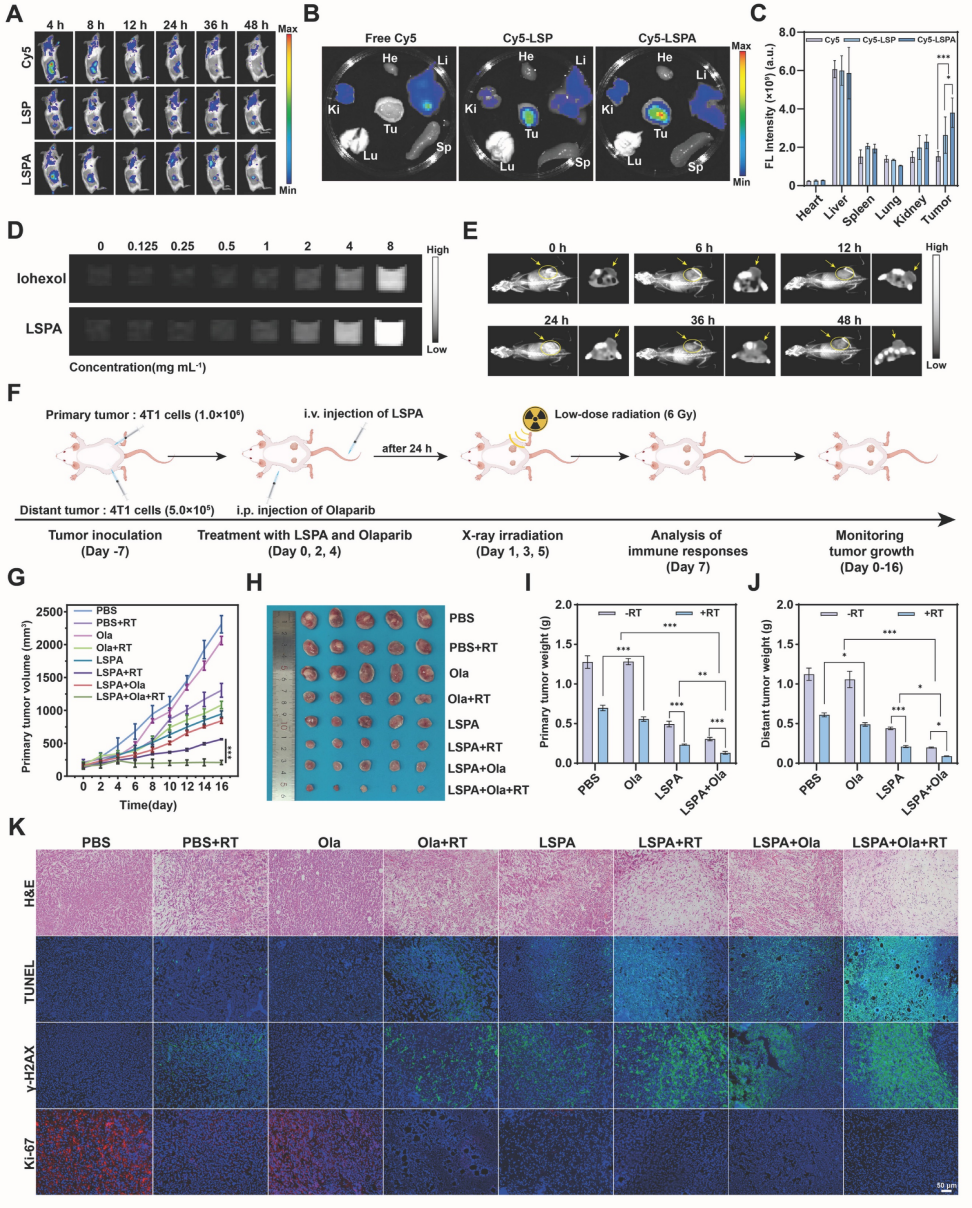
***In vivo* tumor targeting and theranostic efficacy. (A)** *In vivo* fluorescence images of 4T1 tumor-bearing mice at various time points post-injection with free Cy5, Cy5-LSP, or Cy5-LSPA (λ_ex_/λ_em_: 640/670 nm). **(B)** *Ex vivo* fluorescence images of major organs (heart (He), liver (Li), spleen (Sp), lung (Lu), and kidney (Ki)) and tumor (Tu) isolated from 4T1 tumor-bearing mice at 48 h post-injection. **(C)** Quantification of fluorescence intensities in major organs and tumors at 48 h post-injection (*n* = 3). **(D)** CT contrast efficiency (HU) of Iohexol vs. LSPA at equivalent concentrations (0-8 mg mL^-1^). **(E)** CT imaging of tumors post-LSPA injection. Yellow arrows indicate tumor regions. **(F)** Schematic of the *in vivo* antitumor treatment schedule. **(G)** Tumor growth curves of primary tumors in 4T1 tumor-bearing mice over 16 days following the indicated treatments (*n* = 5). **(H)** Photograph of excised primary tumors post-treatment.** (I-J)** Weights of primary and distant tumors following the indicated treatments on day 16 (*n* = 5). **(K)** Histopathological H&E, TUNEL, γ-H2AX, and Ki-67 staining of primary tumor tissues following the indicated treatments. Scale bar: 50 μm. Ola: Olaparib; RT: 6 Gy X-ray irradiation. Data are presented as mean ± SD. *P < 0.05; **P < 0.01; ***P < 0.001.

To translate these findings *in vivo*, we first confirmed the tumor-targeting capability and safety of LSPA. Following intravenous administration in 4T1 tumor-bearing mice, Cy5-labeled LSPA exhibited significantly enhanced tumor accumulation and prolonged retention compared to uncoated LSP or free Cy5, thereby confirming the advantage of the SA coating in enabling passive targeting via the EPR effect (Figure 6A).

*Ex vivo* organ analysis at 48 h post-injection corroborated the superior tumor-specific accumulation of LSPA (Figure 6B-C). Notably, LSPA exhibited excellent hemocompatibility, inducing negligible hemolysis even at high concentrations (Figure S19). Importantly, we leveraged the high-Z of Lu to establish LSPA as a CT contrast agent for image-guided therapy. *In vitro*, LSPA demonstrated concentration-dependent signal enhancement, which was 1.86-fold greater than that of the clinical agent Iohexol (Figure 6D, S20). *In vivo*, CT imaging revealed that LSPA accumulation in the tumor reached its peak at 24 h post-injection and remained elevated at 48 h (Figure 6E).

To evaluate the *in vivo* behavior of LSPA nanoparticles, we initially assessed their biodistribution. Following intravenous administration in tumor-bearing mice, the blood concentration of LSPA was monitored over a 48-h period using ICP-MS for quantification of the Lu element. As shown in Figure S21A, LSPA exhibited a prolonged blood circulation profile, with a calculated circulation half-life (t_1/2_) of approximately 11.75 h, indicating high *in vivo* stability. Subsequently, we evaluated the tissue biodistribution at 48 h post-injection (Figure S21B-G). Notably, LSPA nanoparticles exhibited substantial accumulation in tumor tissue, reaching a concentration of 20.18 ± 0.78 µg g^-1^, which was significantly higher than that in most other tissues, with the exception of reticuloendothelial system (RES) organs. This pronounced tumor uptake can be attributed to the enhanced permeability and retention (EPR) effect. As anticipated for nanoparticles of this size range, considerable accumulation was also observed in the liver (19.05 ± 1.72 µg g^-1^) and spleen (19.12 ± 4.97 µg g^-1^), indicating predominant clearance via the hepatosplenic pathway.

### *In vivo* antitumor efficacy and abscopal effect

Guided by our imaging results, we evaluated the therapeutic efficacy in a bilateral 4T1 tumor model. The combination of LSPA + Olaparib + RT elicited a robust therapeutic response in both irradiated primary and non-irradiated distant (abscopal) tumors, while maintaining stable body weight (Figure 6F, S22). This triple-combination therapy achieved the highest tumor growth inhibition (TGI) in primary tumors (89.70 ± 1.52%), significantly outperforming LSPA + RT (TGI = 81.60 ± 0.60%) (Figure 6G-I, Table S1). Most importantly, this localized treatment triggered a powerful systemic antitumor response, known as the abscopal effect. The triple therapy induced near-complete regression of non-irradiated, distant tumors (TGI = 91.99 ± 0.38%), an effect rarely observed with low-dose radiation alone (Figure 6J, S23-S24). Bioluminescence imaging further confirmed this profound and systemic tumor eradication (Figure S25).

To definitively determine the functional role of the cGAS-STING pathway in mediating the observed therapeutic effects, an *in vivo* inhibition study was conducted using the specific cGAS inhibitor RU.521 42. As shown in Figure S26, co-administration of RU.521 (5 mg kg^-1^) with the combination therapy partially attenuated its antitumor efficacy. Tumor growth inhibition in the RU.521 co-treated group was greater than that in the PBS control group but less pronounced than that in the group receiving combination therapy alone. To validate the molecular mechanism of inhibitor action, the activation of the STING signaling pathway in tumor tissues was assessed. Western blot analysis confirmed that the LSPA + Olaparib + RT regimen robustly induced phosphorylation of TBK1 and IRF3, which was significantly suppressed in the presence of RU.521 (Figure S27). These results provide direct and compelling evidence that the potent antitumor immunity elicited by our radioimmunotherapy approach is mechanistically dependent on cGAS-STING signaling.

Histological analysis of the primary tumors revealed that the triple-combination group caused the most extensive necrosis and apoptosis (H&E and TUNEL staining), the highest levels of DNA damage (γ-H2AX staining), and the greatest reduction in proliferation (Ki-67 staining) (Figure 6K). Similar, albeit less pronounced, effects were observed in the distant tumors, suggesting a systemic immune-mediated antitumor response (Figure S28). Notably, histological examination of major organs and serum biochemistry profiling revealed minimal systemic toxicity, thereby confirming the high biocompatibility and safety of the therapeutic regimen (Figure S29-S30). To further evaluate the long-term safety profile required for clinical translation, a 28-day repeated-dose toxicity study was conducted in healthy mice. Following three intravenous administrations of LSPA over a 28-day period, key serum biomarkers of hepatic function (AST and ALT) and renal function (CRE and BUN) were assessed. No significant elevations in these biomarkers were observed compared to the saline-treated control group, and all measured values remained within normal physiological ranges (Figure S31). These findings demonstrate the favorable long-term safety and biocompatibility of the LSPA platform.

### Synergistic remodeling of the tumor immune microenvironment

**Figure 7:**
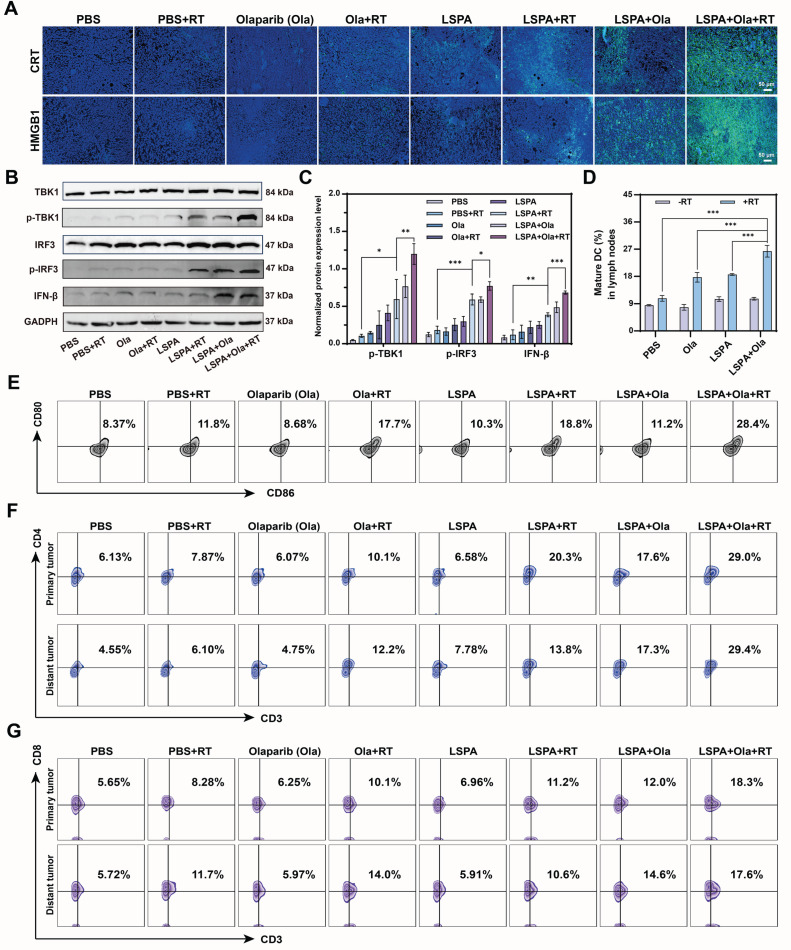
** Synergistic remodeling of the tumor immune microenvironment. (A)** Immunofluorescence staining images of CRT and HMGB1 expression in tumor tissues following the indicated treatments. Scale bar: 50 μm. **(B)** Western blot analysis of cGAS-STING pathway activation biomarkers (p-TBK1, p-IRF3, IFN-β) in tumor lysates. **(C)** Normalized protein expression levels of p-TBK1, p-IRF3, and IFN-β in tumor lysates (*n* = 3). **(D)** Populations of mature DCs (CD11c^+^ CD80^+^ CD86^+^) in tumor-draining lymph nodes (*n* = 3). **(E)** Representative flow cytometry plots of mature DCs in tumor-draining lymph nodes. **(F)** Representative flow cytometry plots showing the percentage of CD4^+^ T helper cells within primary and distant tumors. The CD4^+^ population was gated from the parent CD3^+^ T cell population. **(G)** Representative flow cytometry plots showing the percentage of CD8^+^ cytotoxic T cells within primary and distant tumors. The CD8^+^ population was also gated from the parent CD3^+^ T cell population. Ola: Olaparib; RT: 6 Gy X-ray irradiation. Data are presented as mean ± SD. *P < 0.05; **P < 0.01; ***P < 0.001.

To confirm that the observed *in vivo* efficacy was immune-mediated, we conducted an analysis of the TME. Immunohistochemical staining of primary tumors from the LSPA + Olaparib + RT group showed the highest expression of the ICD biomarkers CRT and HMGB1 (Figure 7A). This corresponded with the strongest activation of the cGAS-STING pathway in tumor lysates, as evidenced by maximal expression of p-TBK1, p-IRF3, and IFN-β (Figure 7B-C).

This local immune activation triggered a systemic antitumor response. DC maturation in tumor-draining lymph nodes was highest in the LSPA + Olaparib + RT group (28.4%), demonstrating efficient immune priming (Figure 7D-E). As a result, there was a substantial infiltration of effector T cells into the TME. Flow cytometry analysis revealed that the LSPA + Olaparib + RT group exhibited the highest proportions of both CD4^+^ helper T cells (29.0%) and CD8^+^ cytotoxic T cells (18.3%) in primary tumors (Figure 7F-G, S32). Notably, this significant T cell infiltration was also observed in distant tumors (CD4^+^: 29.4%; CD8^+^: 17.6%), providing a direct mechanism for the observed abscopal effect 31. Representative flow cytometry analysis shows T cell infiltration in primary and distant tumors, along with the relative percentages of CD4^+^ T helper cells and CD8^+^ cytotoxic T cells. Both populations were gated from the parent CD3^+^ T cell population. Separate analyses are presented to provide a clear quantification of the infiltration of each T cell subset, which serves as a key indicator of the anti-tumor immune response 43-45.

Furthermore, the combination therapy fundamentally reversed immunosuppression within the TME. It induced the most substantial decrease in immunosuppressive regulatory T cells (T_regs_) (Figure S33) and facilitated the repolarization of tumor-associated macrophages (TAMs) toward the antitumor M1 phenotype (Figure S34). This immunologically “hot” TME was characterized by elevated systemic levels of pro-inflammatory cytokines, including IFN-β, IL-6, IFN-γ, and TNF-α (Figure S35-S36), thereby confirming the induction of a robust and systemic antitumor immune response.

### Induction of long-term immune memory and tumor recurrence prevention

**Figure 8:**
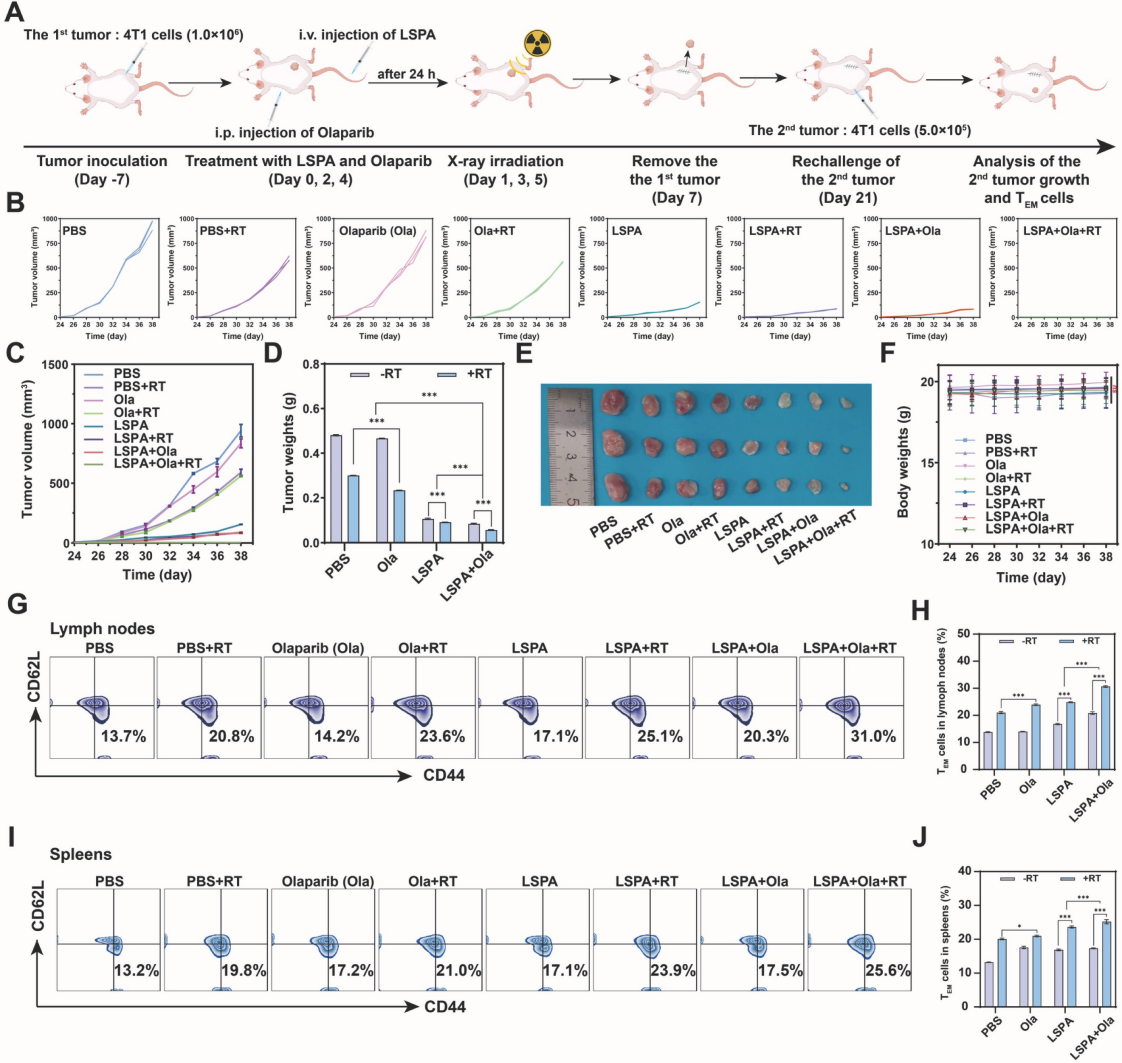
** Induction of long-term immune memory and tumor recurrence prevention. (A)** Schematic of the tumor re-challenge experiment. **(B-C)** Individual and combined growth curves of the 2^nd^ tumors post-rechallenge within 38 days (*n* = 3). **(D)** Weights of the 2^nd^ tumors on day 38 post-rechallenge (*n* = 3).** (E)** Photographs of the excised 2^nd^ tumors on day 38 post-rechallenge. **(F)** Body weight changes of the tumor rechallenged mice from day 24 to day 38 (*n* = 3). **(G)** Representative flow cytometry plots of effector memory T cells (T_EM_ cells, CD3^+^ CD44^+^ CD62L^-^) in lymph nodes on day 38 post-rechallenge. **(H)** Proportions of T_EM_ cells in lymph nodes (*n* = 3). **(I)** Representative flow cytometry plots of T_EM_ cells in spleens on day 38 post-rechallenge. **(J)** Proportions of T_EM_ cells in spleens (*n* = 3). Ola: Olaparib; RT: 6 Gy X-ray irradiation. Data are presented as mean ± SD, *ns*: no significance; *P < 0.05; **P < 0.01; ***P < 0.001.

Finally, we addressed a significant clinical challenge in TNBC: the prevention of tumor recurrence. In a tumor rechallenge model, mice that had previously achieved complete tumor clearance through the LSPA + Olaparib + RT treatment demonstrated long-term resistance to secondary tumor inoculation (Figure 8A-E). In contrast, control groups exhibited rapid secondary tumor growth. Treatments were well-tolerated with no significant body weight changes throughout the study (Figure 8F).

This durable protective effect was mechanistically supported by the development of robust immunological memory. Flow cytometric analysis revealed that the LSPA + Olaparib + RT group had the highest proportions of T effector memory (T_EM_) cells (CD3^+^ CD44^+^ CD62L^-^) in both the lymph nodes (31.0%) and spleen (25.6%) (Figure 8G-J). These findings demonstrate that our synergistic theranostic strategy not only eradicates established primary and metastatic tumors but also establishes durable immune surveillance capable of preventing future recurrence, thereby providing a potential avenue toward curative intervention.

## Discussion

Although RT is a cornerstone in the management of TNBC, its efficacy is often limited by radioresistance and an immunosuppressive TME, which necessitates high radiation doses (≥50 Gy) that risk significant toxicity 2,3. This clinical dilemma highlights the need for new approaches. Our study proposes a novel low-dose (6 Gy) radioimmunotherapy that challenges this paradigm. By combining a rationally designed LSPA nanosensitizer with the PARP inhibitor Olaparib, we transform a low radiation dose into a powerful trigger for systemic and durable antitumor immunity.

The rational design of the LSPA theranostic nanosensitizer is a pivotal component of our approach. The choice of Lu was guided by systematic screening, which identified Lu^3+^ as the most potent ROS generator among the lanthanides tested under X-ray irradiation (Figure 2A, and S1). Although the chelation-driven assembly is derived from established principles 46, our LSPA nanoplatform represents a significant advancement. The specific coordination of Lu^3+^ with Sal^-^ within a PVP/SA-stabilized nanostructure is novel and provides distinct advantages. LSPA nanoparticles demonstrate excellent colloidal stability and pH-responsive Lu^3+^/Sal^-^ release in the acidic TME (Figure 2H-K), thereby enhancing therapeutic efficacy while minimizing systemic X-ray exposure 16. This optimal Lu^3+^: Sal^-^ molar ratio was determined after screening a range from 1:16 to 1:128, as the 1:64 ratio provided the best balance between high colloidal stability, as measured by DLS, and maximal salicylate encapsulation efficiency. Functionally, this formulation also demonstrated superior payload retention at physiological pH and the most potent radiosensitization under 6 Gy irradiation. The concentration of the PVP stabilizer was also optimized; 1 mg mL^-1^ was selected as it yielded a narrow particle size distribution (PDI: 0.36 ± 0.02) and good stability prior to surface coating.

Furthermore, Sal^-^ serves a dual function: beyond structural stabilization, it acts as a bioactive molecule upon release, inducing mild oxidative stress 35 SA surface functionalization improves biocompatibility and tumor targeting via the EPR effect (Figure 6A-C) 30. Notably,* in vitro* assays demonstrated that LSPA mediates substantial radiosensitization in 4T1 cells at 6 Gy X-rays, as evidenced by an SER of 1.55 (Figure 3M) and elevated ROS production (Figure 3N-P and S11). This capability to achieve radiosensitization at dramatically reduced doses compared to conventional RT (≥ 50 Gy) represents a critical step toward decoupling immunogenic efficacy from dose intensity 5.

The selection of Olaparib was a deliberate decision. As the first-in-class PARP inhibitor, Olaparib is supported by extensive clinical validation and possesses a well-characterized mechanism of action that demonstrates synergy with radiotherapy, particularly through its capacity to enhance DNA damage and activate the cGAS-STING pathway 47. Its established preclinical dosing protocols also ensured experimental reproducibility 48. These factors made it the ideal candidate for this proof-of-concept study.

The synergy between LSPA-mediated radiosensitization and PARP inhibition is fundamental to the therapeutic efficacy of our strategy. While each component is individually beneficial, their combination induces a level of DNA damage that neither can achieve alone at a low radiation dose. Olaparib potentiates LSPA-enhanced RT by blocking DNA repair pathways 19,20 This synergy was evidenced by increased γ-H2AX fluorescence intensity, extended comet tail lengths, mitochondrial dysfunction, and apoptosis in 4T1 cells receiving the triple-combination therapy (Figure 4, S13-17). By achieving this at a 6 Gy dose, our strategy offers a significant dose-sparing advantage. This overwhelming genotoxic stress provides a direct mechanistic link to immune activation. The extensive DNA fragmentation (Figure 4G-H) generates cytosolic DNA fragments, which are canonical ligands for the cGAS sensor 49. This, in turn, resulted in robust activation of the downstream STING pathway (p-TBK1, p-IRF3, and IFN-β) in both immune cells (Figure 5D-E) and tumors (Figure 7B-C). Potent STING activation correlated with biomarkers of ICD, including CRT exposure and the release of ATP and HMGB1 (Figure 5A-C, and 7A). As a result, DC maturation, a critical step in the initiation of adaptive immunity 50, was significantly enhanced (Figure 5F-G, and 7D-E).

The STING-mediated immune activation reprogrammed the immunosuppressive TME, a key obstacle in TNBC RT 51. It is essential to interpret the 6 Gy radiation dose used in our study. Although higher than a single fraction in conventional RT, it represents a clinically relevant, sub-curative dose frequently used in hypofractionated regimens such as stereotactic body radiation therapy (SBRT). Importantly, our strategy demonstrates that such a single, manageable dose, when combined with our nanoplatform, is sufficient to elicit a robust systemic immune response. This establishes a paradigm for utilizing radiation not only for its direct cytotoxic effects, but also as a potent *in situ* vaccine primer.

The resulting immune cascade was both potent and comprehensive. Combination therapy with LSPA + Olaparib + RT significantly increased CD4^+^ and CD8^+^ T cell infiltration in primary and distant tumors (Figure 7F-G, and S32), indicating a systemic cytotoxic T cell response consistent with STING activation, which enhances T cell recruitment 52. This combination therapy also reduced T_reg_ cell infiltration (Figure S33) and repolarized TAMs from the M2 to the M1 phenotype (Figure 5H-K, and S34), thereby mitigating immunosuppression 53. The elevated systemic pro-inflammatory cytokines, including IFN-β, IL-6, IFN-γ, and TNF-α (Figure S36), further confirmed robust systemic antitumor immune responses. These responses significantly inhibited the growth of irradiated primary and non-irradiated distant (abscopal) tumors (Figure 6G-J, S23-25, and Table S1), demonstrating the conversion of localized treatment into a body-wide therapeutic effect.

Our strategy demonstrates that radiation can be used not only for its direct cytotoxic effects but also as a potent *in situ* vaccine primer. Our findings demonstrate that the robust immune activation is sustained rather than transient. The LSPA + Olaparib + RT regimen effectively abrogated 4T1 tumor recurrence and significantly increased T_EM_ cell proportions in lymphoid organs (Figure 8G-J). These findings suggest that the treatment establishes robust immune surveillance capable of preventing relapse, offering a promising approach to improve long-term TNBC survival. Additionally, LSPA nanoparticles also exhibited excellent CT imaging capabilities (Figure 6D-E, and S20), providing a valuable theranostic advantage for non-invasive monitoring and image-guided therapy. To contextualize our work within the existing radioimmunotherapy strategies, we have compared our LSPA-Olaparib strategy with other state-of-the-art radioimmunotherapy approaches, and summarized the key findings in Table S2. This comparison highlights several unique advantages of our system in achieving the goal of enhancing the efficacy of low-dose radioimmunotherapy while limiting systemic toxicity.

Compared to other nanosensitizers, the Lu core shows higher photoelectric absorption and electron yield, leading to stronger ROS amplification under clinically relevant irradiation. Additionally, our system releases Sal^-^ specifically in the TME, enhancing radiosensitivity by reducing redox buffering and DNA damage tolerance where radiation energy is deposited. Unlike PARPi-RT, which depends on systemic PARP inhibition and specific DDR conditions, Lu-Sal uses localized ROS and controlled Sal^-^ release, minimizing off-target effects and simplifying combination with RT. These features together form a dual-axis radiosensitization approach not possible with existing high-Z nanosensitizers or PARPi combinations.

Despite promising results, this study has several limitations that must be addressed to facilitate clinical translation. First, the murine 4T1 orthotopic syngeneic TNBC model is useful for studying radiosensitization in an intact immune environment, but it does not fully reflect human TNBC heterogeneity or patient-specific stromal and vascular barriers. To improve translational value, future studies should use more advanced models such as (i) orthotopic patient-derived xenografts (PDX) to better understand nanoparticle transport and drug release in human tumors, and (ii) humanized models to capture interactions between radiation, nanoparticles, and the immune system. *Ex vivo* tumor slices and organoids can also be used for rapid testing to support data on efficacy, biodistribution, and safety. Second, long-term pharmacokinetic and toxicity studies of LSPA are needed, even though Lu-based agents generally have good safety profiles 54,55. Future research should also focus on optimizing the LSPA formulation for large-scale production to facilitate clinical translation. Finally, further study of how STING activation interacts with other immune pathways could guide combination therapies and extend this approach to other “cold” tumors.

## Conclusion

In conclusion, we have developed a clinically viable and multifunctional low-dose (6 Gy) radioimmunotherapy strategy that synergistically integrates a pH-responsive organolutetium theranostic nanosensitizer (LSPA) with the PARP inhibitor Olaparib. This dual-action system successfully achieves a critical goal in modern oncology: dissociating immunogenic potency from high-dose radiation toxicity, thereby enabling potent cGAS-STING-mediated innate and adaptive immune activation while minimizing off-target effects. The integration of CT imaging capability provides a theranostic advantage, allowing for non-invasive guidance and monitoring. In addition to substantial suppression of both primary and metastatic tumors, the treatment induces long-lasting immunological memory and effective protection against tumor recurrence, addressing a longstanding unmet clinical need in the management of TNBC. These findings not only establish the LSPA-Olaparib combination as a promising approach for *in situ* vaccine priming but also provide a versatile and powerful platform for the development of next-generation nano-enabled radioimmunotherapies targeting a wide range of immunologically “cold” tumors.

## Materials and Methods

### Materials

Lutetium (III) chloride hexahydrate (LuCl_3_·6H_2_O), sodium salicylate (NaSal), PVP (K30), mouse serum albumin (SA), amino-functionalized Cy5 dye (Cy5-NH_2_) and other chemical reagents (analytical grade) were purchased from Aladdin (Shanghai, China). The CCK-8 assay kit, comet assay kit, γ-H2AX immunofluorescence-based DNA damage detection kit, calcein-AM/PI cell viability and cytotoxicity assay kit, mouse HMGB1 ELISA kit, ATP detection kit and ROS assay kits were obtained from Beyotime (Shanghai, China). The BBoxiProbe O22 and BBoxiProbe O27 probes were obtained from Bestbio (Shanghai, China). Cell culture and processing reagents, including 4',6-diamidino-2-phenylindole (DAPI), 4% paraformaldehyde, radioimmunoprecipitation assay (RIPA) lysis buffer, and phenylmethanesulfonyl fluoride (PMSF), were acquired from Solarbio (Beijing, China). Primary antibodies for Western blotting and immunofluorescence analysis, including rabbit anti-phospho-TBK1, rabbit anti-phospho-IRF3, rabbit anti-IRF3, rabbit anti-CD44, rabbit anti-HMGB1, mouse anti-GAPDH, and mouse anti-Calreticulin were supplied by Bioss (Beijing, China). For flow cytometric analysis, the following fluorochrome-conjugated antibodies were used: anti-CD206-APC, anti-CD8-PE, anti-CD4-APC, anti-CD3-FITC, anti-CD80-APC, anti-CD86-PE, anti-CD11c-FITC, anti-F4/80-FITC, and anti-Foxp3-PE, all of which were obtained from Invitrogen (USA). Recombinant mouse IL-4 protein was also from Invitrogen. Millipore Milli-Q ultrapure water (18.2 MΩ cm; USA) was used throughout all experimental procedures. All other chemical reagents were of analytical grade and used as received without further purification.

### Cell culture and animal models

4T1, NIH 3T3 fibroblast, and RAW 264.7 macrophage cell lines were obtained from the American Type Culture Collection (ATCC, USA). The DC2.4 dendritic cell line was from Kanglang Biological Technology (Shanghai, China). Luciferase-expressing 4T1 (4T1-Luc) cells were acquired from PerkinElmer, Inc. (USA). 4T1, 4T1-Luc, and DC2.4 cells were cultured in RPMI 1640 medium. NIH 3T3 and RAW 264.7 cells were cultured in DMEM. All media were supplemented with 10% FBS, 100 U mL⁻¹ penicillin, and 100 µg mL^-1^ streptomycin. Cells were maintained at 37 °C in a humidified atmosphere with 5% CO_2_. Female BALB/c mice (6-8 weeks old, 18-20 g) were purchased from Yaokang Biotechnology (Guangzhou, China). Tumor volumes and body weights were recorded on a daily basis throughout the duration of the study. All procedures were carried out in full compliance with institutional guidelines, and animals were closely monitored to ensure that tumor burden remained within the maximum permissible limits.

### Preparation and characterization of LSPA nanoparticles

LSP nanoparticles (Lu^3+^/Sal^-^ molar ratio of 1:64) were prepared by mixing 2 mL of NaSal solution (6.4 M) with 2 mL of LuCl_3_ solution (50 mM) under magnetic stirring for 2 h at 4 °C. Subsequently, 2 mL of PVP solution (1 mg mL^-1^) was added, and the mixture was stirred for 10 min, followed by probe sonication (200 W, 3 s on/4 s off cycle, 10 min). The resulting LSP nanoparticles were collected by centrifugation (5,000 rpm, 15 min) and washed three times with deionized water. For LSPA preparation, 24 mg of LSP was dispersed in 20 mL of mouse serum albumin solution (165 µg mL^-1^) and stirred for 2 h at 4 °C. The resulting LSPA nanoparticles were collected by centrifugation (5,000 rpm, 15 min) and washed three times with deionized water. To assess morphological stability, LSPA nanoparticles were incubated for 24 h at 37 °C in RPMI 1640 medium supplemented with 10% FBS and 10 µg mL^-1^ heparin. After incubation, the morphology of the nanoparticles was examined by TEM.

Hydrodynamic diameter, PDI, and zeta potential were measured using dynamic light scattering (DLS) on a Malvern Zetasizer Ultra (UK). Morphology and elemental composition were analyzed by TEM (JEM-F200, JEOL, Japan). Chemical structure was verified by FT-IR spectroscopy (Nicolet iS50, Thermo Fisher, USA) and XPS (AXIS Supra+, Shimadzu, Japan). Sal^-^ encapsulation was determined by measuring supernatant absorbance at 296 nm. Lu^3+^ content was measured by ICP-MS (Agilent 7900, USA). Colloidal stability was assessed by monitoring hydrodynamic size changes in water, PBS, and 10% FBS over 48 h. To prepare Cy5-labeled LSP and LSPA nanoparticles for* in vivo* imaging, a chelation-based method was employed. During the synthesis of LSP and LSPA nanoparticles, Cy5-NH_2_ was introduced at a low feed ratio (typically 1.0 % relative to total Lu^3+^ in the dispersion). The mixture was then allowed to react for 2 h at room temperature in the dark under gentle shaking to promote the chelation between Cy5-NH_2_ and Lu^3+^. The remaining preparation procedure was performed as described above. The labeled nanoparticles were purified through three cycles of centrifugation (5,000 rpm, 15 min) and washed with deionized water.

### pH-responsive release study

LSPA nanoparticles (600 µg mL^-1^) were dispersed in PBS buffer (10 mM, pH 7.4) or acetate buffer (10 mM, pH 4.8) and incubated at 37 °C with shaking (300 rpm). At designated time points, aliquots were centrifuged, and the supernatants were analyzed for Sal^-^ (UV-Vis at 296 nm) and Lu^3+^ (ICP-MS) content (*n* = 3). The cumulative release was calculated using the standard formula accounting for sample withdrawal 56.

### *In vitro* therapeutic efficacy and synergy analysis

Cell viability was assessed using the CCK-8 assay after 24 h of treatment. The sensitizer enhancement ratio (SER) was determined by pre-treating 4T1 cells with LSPA (600 µg mL^-1^) for 6 h, followed by irradiation with varying X-ray doses (0-6 Gy). IC_50_ values were calculated from cell viability data to determine the SER. The SER was derived using the following equation 57,58:


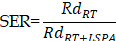


where *Rd_RT_* represents the radiation dose (6.48 Gy) required to achieve 50% cell viability, and *Rd_RT +LSPA_* is the radiation dose (4.18 Gy) required to reach 50% cell viability when LSPA (600 µg mL^-1^) is added.

For synergy analysis, 4T1 cells were treated with a matrix of LSPA (75-1200 µg mL^-1^) and Olaparib (12.5-200 µg mL^-1^) concentrations, with or without 6 Gy irradiation. Synergy scores were calculated using the SynergyFinder web application (ZIP model) 36. Live/dead cell visualization was performed with Calcein-AM/PI staining. The long-term impact on cell proliferation was evaluated using a colony formation assay, where treated cells were cultured for 14 days before colonies were fixed and stained with crystal violet.

### *In vitro* radiosensitization and ROS detection

The radiosensitizing effect was evaluated in cell-free assays by measuring the degradation of DPPH (517 nm), DPBF (for ^1^O_2_, 410 nm), and MB (for •OH, 665 nm) after exposure to X-rays (6 Gy, RAD SOURCE RS2000, USA). ROS generation was further confirmed using ESR spectroscopy with TEMP (for ^1^O_2_) and DMPO (for •OH) as spin traps. For cellular assays, 4T1 cells were treated with LSPA (600 µg mL^-1^) for 6 h, with or without subsequent 6 Gy irradiation. Intracellular ROS was detected using the DCFH-DA probe, BBoxiProbe O22 probe (for ^1^O_2_), and BBoxiProbe O27 probe (for •OH) through confocal laser scanning microscopy (LSM880, ZEISS, Germany) and flow cytometry (Accuri C6, BD Biosciences, USA).

### Mechanistic *in vitro* analyses

DNA damage was assessed by immunofluorescence staining for γ-H2AX foci and by single-cell gel electrophoresis (Comet assay). Mitochondrial membrane potential was evaluated using a JC-1 probe, with the shift from red (J-aggregates) to green (J-monomers) fluorescence indicating depolarization. To assess ICD, surface CRT exposure was detected by immunofluorescence, while intracellular ATP and HMGB1 levels were quantified using commercial ELISA kits according to the manufacturer's instructions.

### *In vitro* immune activation assays

To generate conditioned medium (CM), 4T1 cells were subjected to the various treatments for 24 h. Subsequently, DC2.4 cells were incubated with the resulting CM for 12 h (for Western blot analysis) or 24 h (for maturation assay). Activation of the cGAS-STING pathway (p-TBK1, p-IRF3, IFN-β) was analyzed by Western blotting. DC maturation was assessed via flow cytometric analysis of surface markers CD80 and CD86. For macrophage repolarization, RAW264.7 cells were initially polarized to the M2 phenotype using murine IL-4 (25 ng mL^-1^) for 12 h, followed by culture in CM for 24 h. Repolarization was determined by flow cytometry based on the expression of M1 (CD86^+^) and M2 (CD206^+^) phenotypic markers.

### Scratch wound healing assays

The effect on cell migration was assessed via a wound healing assay. A scratch was made in a confluent monolayer of 4T1 cells, which were then subjected to the various treatments. Wound closure was imaged at 0 and 24 h. The percentages of wound closure were calculated by the following equation:
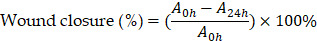


where *A_0h_* is the initial wound area, *A_24h_* is the wound area after 24 h of the initial scratch, both in µm^2^.

### Hemolysis assay

Fresh murine RBCs were incubated with varying concentrations of LSPA (75-1200 µg mL^-1^) for 3 h at 37 °C. After centrifugation, the absorbance of the supernatant was measured at 415 nm to quantify hemoglobin release. RBCs in PBS and deionized water served as negative and positive controls, respectively.

### *In vivo* studies

Bilateral subcutaneous 4T1 tumor models were established by inoculating 1×10^6^ cells into the right flank (as the primary tumor) and 5×10^5^ cells into the left flank (as the distant tumor). For biodistribution analysis, mice were intravenously administered Cy5-labeled LSPA (4.8 mg kg^-1^) and imaged at various time points using an IVIS Spectrum imaging system (PerkinElmer, USA). For CT imaging, mice received LSPA (4.8 mg kg^-1^) and were scanned using a GE Discovery CT750 HD scanner.

For evaluation of antitumor efficacy (*n* = 5 per group), mice were treated with LSPA (42 mg kg^-1^, intravenous) and/or Olaparib (50 mg kg^-1^, intraperitoneal) on days 0, 2, and 4. The LSPA dosage was selected based on preliminary dose-optimization studies aimed at achieving maximal therapeutic efficacy with minimal toxicity, while the Olaparib dosage was determined according to previously published protocols 48. Primary tumors were subjected to 6 Gy irradiation (RAD SOURCE X-ray RS2000, USA) on days 1, 3, and 5. Tumor volumes and body weights were recorded throughout the study. At the experimental endpoint, tumors and major organs were collected and processed for organ weight measurement, histopathological analysis (H&E, TUNEL, γ-H2AX, Ki-67), and immunological analysis. Serum was collected for biochemical analysis (ALT, AST, CRE, BUN) and cytokine quantification (ELISA). Tumor growth inhibition (TGI) values were calculated using the following equation:


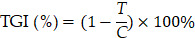


where *T* is the average tumor volume of the treatment groups, *C* is the average tumor volume of the control (PBS) group. To investigate the role of the cGAS-STING pathway, mice in the inhibitor group received RU.521 (5 mg kg^-1^, i.p.) 1 h prior to each radiotherapy session on days 1, 3, and 5, along with concurrent administration of LSPA and Olaparib.

To assess long-term toxicity, healthy BALB/c mice (*n* = 3 per group) received intravenous administrations of LSPA nanoparticles (42 mg kg⁻¹ in 100 µL PBS) or an equivalent volume of PBS once every seven days for a total of three doses. On day 28, blood samples were collected via cardiac puncture, and serum was separated by centrifugation. The levels of ALT, AST, CRE, and BUN were measured using commercial assay kits according to the manufacturer's instructions.

### *In vivo* immune response and memory evaluation

On day 16 post-treatment, tumor-draining lymph nodes and tumors were collected (*n*=3 per group). DC maturation (CD11c⁺ CD80⁺ CD86⁺) in lymph nodes was assessed, along with the analysis of tumor-infiltrating lymphocytes (CD3⁺, CD4⁺, CD8⁺), T_regs_ (CD3⁺ CD4⁺ Foxp3⁺), and TAMs (F4/80⁺ CD86⁺ CD206⁺) in tumors using flow cytometry. Activation of the cGAS-STING pathway in tumor tissue was assessed by Western blot, and serum cytokine levels (IL-10, IL-12, TNF-α, IL-6, IFN-β, IFN-γ) were quantified by ELISA.

For the immune memory study (*n*=3 per group), primary tumors were surgically removed on day 7 post-treatment. On day 21, mice were rechallenged with 1×10^6^ 4T1 cells injected into the contralateral flank. On day 38, splenocytes and lymphocytes were analyzed for T effector memory cells (T_EM_; CD3⁺ CD44⁺ CD62L^-^) by flow cytometry.

### Statistical analysis

All quantitative data are presented as mean ± standard deviation (SD). Statistical comparisons between two groups were performed using an unpaired two-tailed Student's t-test. Comparisons among multiple groups were performed using one-way or two-way analysis of variance (ANOVA) followed by Tukey's post-hoc test for multiple comparisons. All analyses were performed using GraphPad Prism 9.0. A *p*-value < 0.05 was considered statistically significant (*P < 0.05, **P < 0.01, ***P < 0.001).

## Supplementary Material

Supplementary figures.

## Abbreviations

^1^O_2_: Singlet oxygen
•OH: Hydroxyl radical
ALT: Alanine aminotransferase
ANOVA: Analysis of variance
APC: Allophycocyanin
AST: Aspartate aminotransferase
ATP: Adenosine triphosphate
BUN: Blood urea nitrogen
CCK-8: Cell Counting Kit-8
cGAS: cyclic GMP-AMP synthase
CLSM: Confocal laser scanning microscopy
CM: Conditioned medium
CRE: Creatinine
CRT: Calreticulin
CT: Computed tomography
DAPI: 4′,6-diamidino-2-phenylindole
DCFH-DA: 2′,7′-dichlorodihydrofluorescein diacetate
DLS: Dynamic light scattering
DMPO: 5,5-dimethyl-1-pyrroline N-oxide
DPBF: 1,3-diphenylisobenzofuran
DPPH: 2,2-diphenyl-1-picrylhydrazyl
DSBs: DNA double-strand breaks
EE: Encapsulation efficiency
EPR: Enhanced permeability and retention
ESR: Electron spin resonance
FBS: Fetal bovine serum
FT-IR: Fourier-transform infrared
GAPDH: Glyceraldehyde-3-phosphate dehydrogenase
H&E: Hematoxylin and eosin
High-Z: High-atomic-number
HMGB1: High-mobility group box 1
ICD: Immunogenic cell death
IC_50_: Half maximal inhibitory concentration
ICP-MS: Inductively coupled plasma mass spectrometry
IFN: Interferon
IL: Interleukin
IRF3: Interferon regulatory factor 3
JC-1: 5,5′,6,6′-tetrachloro-1,1′,3,3′-tetraethyl-benzimidazolylcarbocyanine iodide
LSP: Lutetium-salicylate-PVP nanoparticles
LSPA: Lutetium-salicylate-PVP-albumin nanoparticles
Lu: Lutetium
MB: Methylene blue
MMP: Mitochondrial membrane potential
NaSal: Sodium salicylate
Ola: Olaparib
PARP: Poly(ADP-ribose) polymerase
PDI: Polydispersity index
PE: Phycoerythrin
PMSF: Phenylmethanesulfonyl fluoride
PVP: Polyvinylpyrrolidone
RBCs: Red blood cells
RIPA: Radioimmunoprecipitation assay
ROS: Reactive oxygen species
RT: Radiotherapy
SA: Serum albumin
Sal^-^: Salicylate
SBRT: Stereotactic body radiation therapy
SD: Standard deviation
SER: Sensitizer enhancement ratio
STING: Stimulator of interferon genes
TAMs: Tumor-associated macrophages
TBK1: TANK-binding kinase 1
TEM (cells): T effector memory cells
TEM (microscopy): Transmission electron microscopy
TEMP: 2,2,6,6-tetramethylpiperidine
TGI: Tumor growth inhibition
TME: Tumor microenvironment
TNBC: Triple-negative breast cancer
TNF-α: Tumor necrosis factor-alpha
Tregs: Regulatory T cells
UV-Vis: Ultraviolet-visible spectroscopy
XPS: X-ray photoelectron spectroscopy
ZIP: Zero interaction potency
γ-H2AX: Phosphorylated H2A histone family member X
