# Supplementary material for: An organolutetium nanosensitizer synergizes with PARP inhibition to unleash STING-mediated immunity for low-dose radioimmunotherapy
Source: Theranostics. 2026 Jan 1;16(4):1720–39. doi: 10.7150/thno.124034 (PMC12680527; doi:10.7150/thno.124034)
Supplement: Supplementary file 1 — Supplementary figures. [file thnov16p1720s1.pdf]

Supplementary Materials

An organolutetium nanosensitizer synergizes with PARP inhibition to unleash STING-mediated immunity for low-dose radioimmunotherapy

Bingchun Zeng [1,2 #], Kai Ling [1,2,3 # *], Qingpeng Yuan [1,2], Zeyang Chen [1,2], Guangrong Zhang [1,2], Wenyue Kang [2], Xuanjun Zheng [2], Chuanghong Liao [2], Youqing Mai [2], Zhongjie Huang [4], Ruibin Huang [3], Tiantian Zhai [1] & Hongyan Jiang [1,2,3,5 *]

[1] Department of Radiation Oncology, Cancer Hospital of Shantou University Medical College, Shantou 515041, China

[2] Department of Pharmacology, Shantou University Medical College, Shantou 515041, China.

[3] Department of Radiology, The First Affiliated Hospital of Shantou University Medical College, Shantou 515041, China.

[4] Department of Radiology, Shenzhen Maternity and Child Health Care Hospital, Shenzhen 518100, China

[5] Department of Thyroid, Breast and Hernia Surgery, General Surgery, The Second Affiliated Hospital of Shantou University Medical College, Shantou 515041, China.

[#] These authors contributed equally to this work

[*]Corresponding author: kailing@stu.edu.cn (ORCID: 0000-0001-9725-926X);

hyjiang@stu.edu.cn (ORCID: 0000-0003-3259-1936)

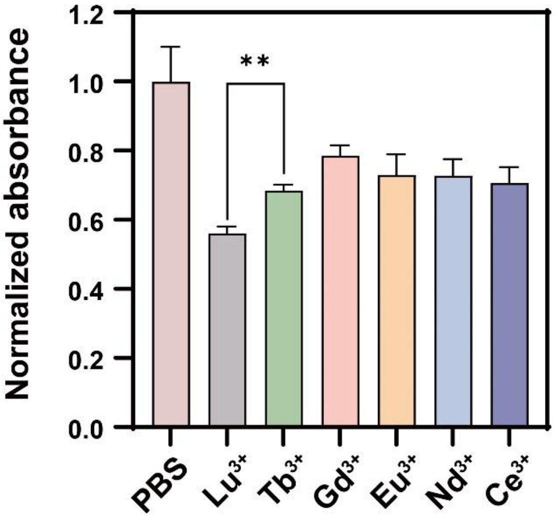

Figure S1. Normalized absorbance of DPPH radicals in the presence of various lanthanide ions following 6 Gy X-ray irradiation (*n* = 3). Data are presented as mean ± SD; $^{**}P < 0.01$.

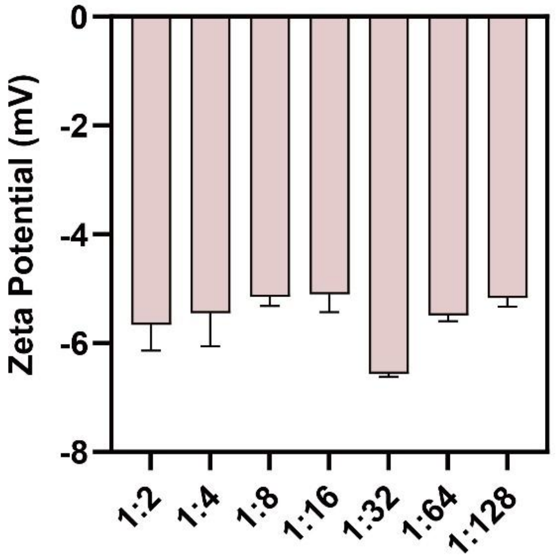

Figure S2. Zeta potential of LSP nanoparticles prepared at varying molar ratios of Lu^{3+}/Sal^{-} (*n* = 3). Data are presented as mean ± SD.

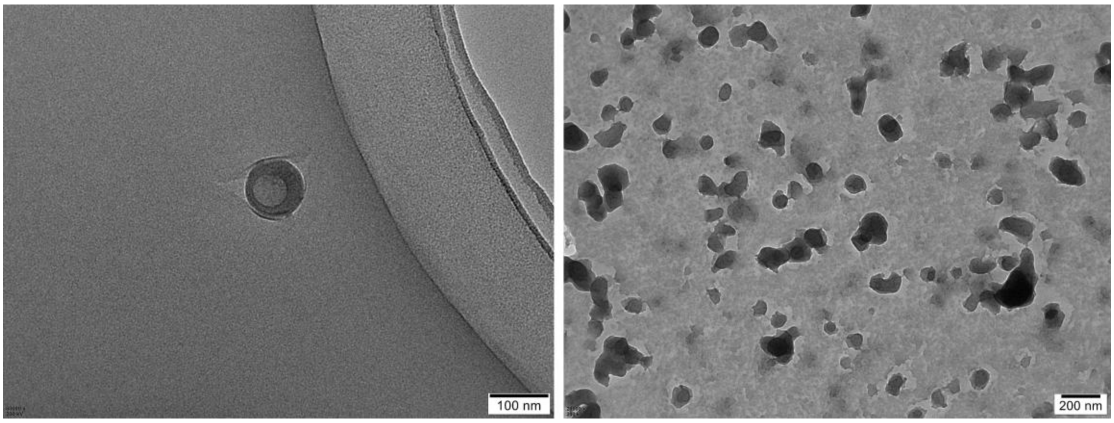

Figure S3. Representative TEM images of LSP nanoparticles.

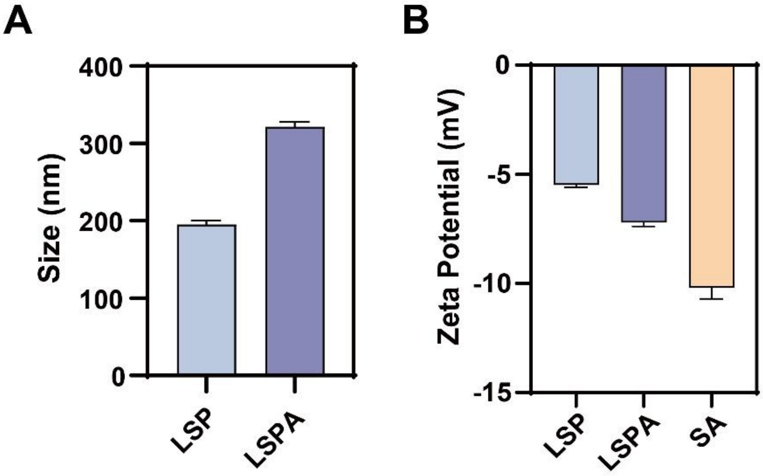

Figure S4. (A) Hydrodynamic size distribution and **(B)** zeta potential of LSP and LSPA (*n* = 3). Data are presented as mean ± SD.

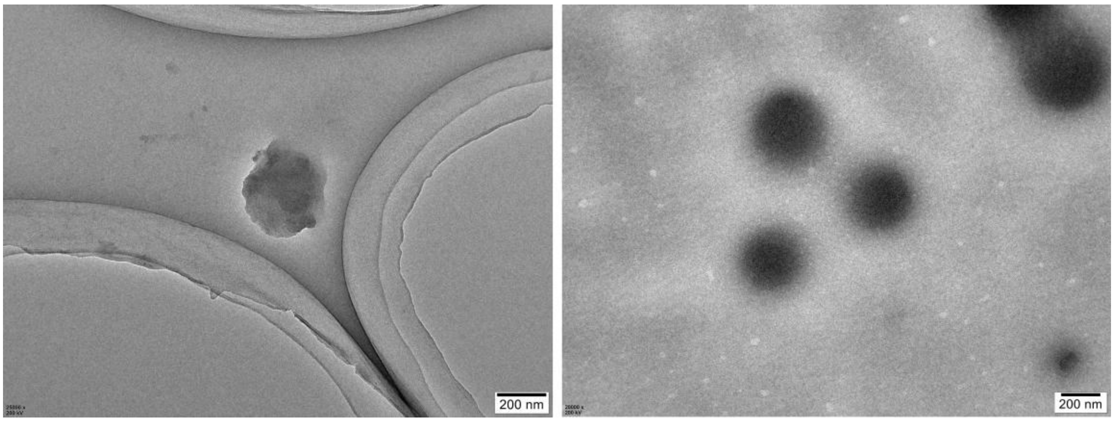

Figure S5. Representative TEM images of LSPA nanoparticles.

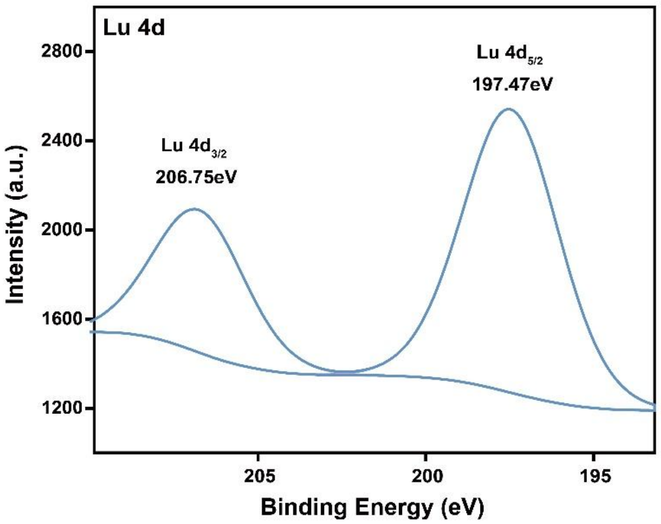

Figure S6. High-resolution XPS spectrum of Lu^{3+} in LSPA nanoparticles.

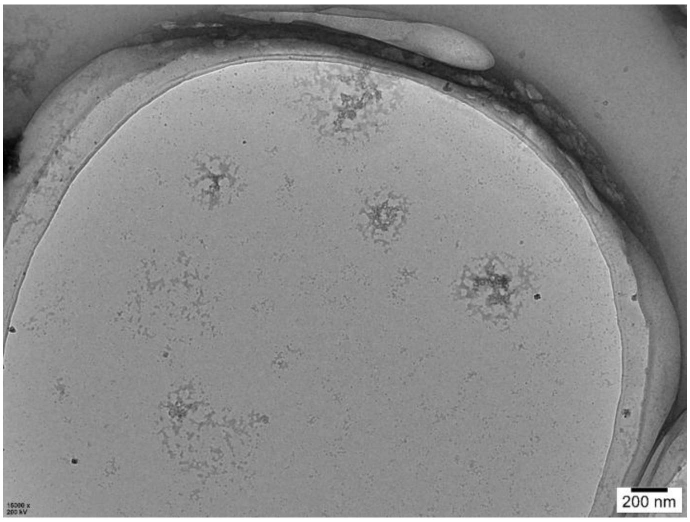

Figure S7. Representative TEM images of LSPA nanoparticle disassembly under pH 4.8 conditions.

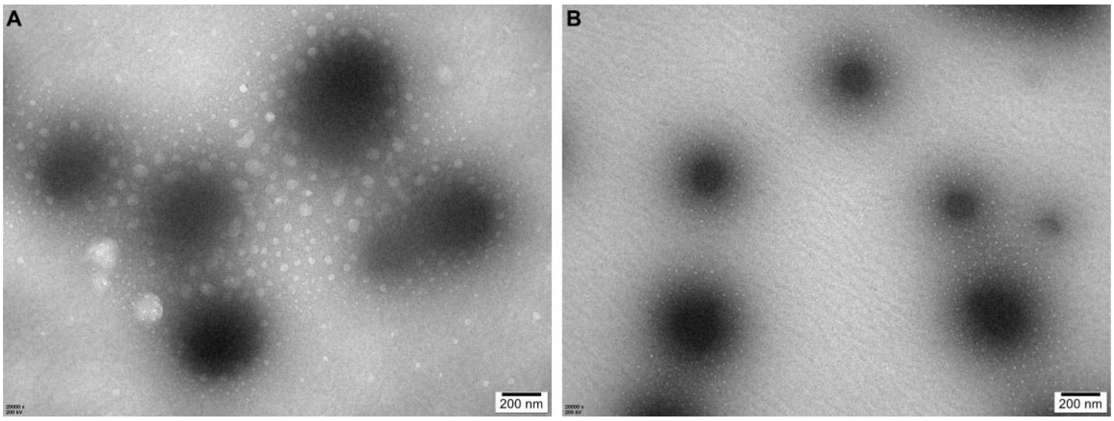

Figure S8. Representative TEM images of LSPA nanoparticles after incubation for 24 h in cell culture media containing 10% FBS (A) or 10 μg mL^{-1} heparin (B).

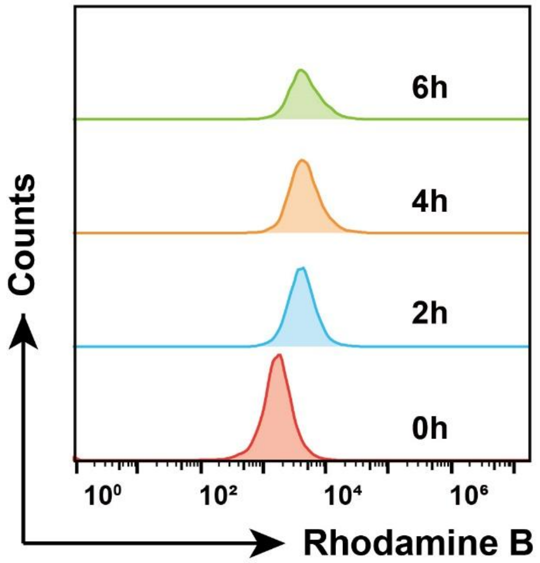

Figure S9. Flow cytometric analysis of 4T1 cells incubated with Rhodamine B-labeled LSPA nanoparticles (600 μg mL^{-1}) at various time points.

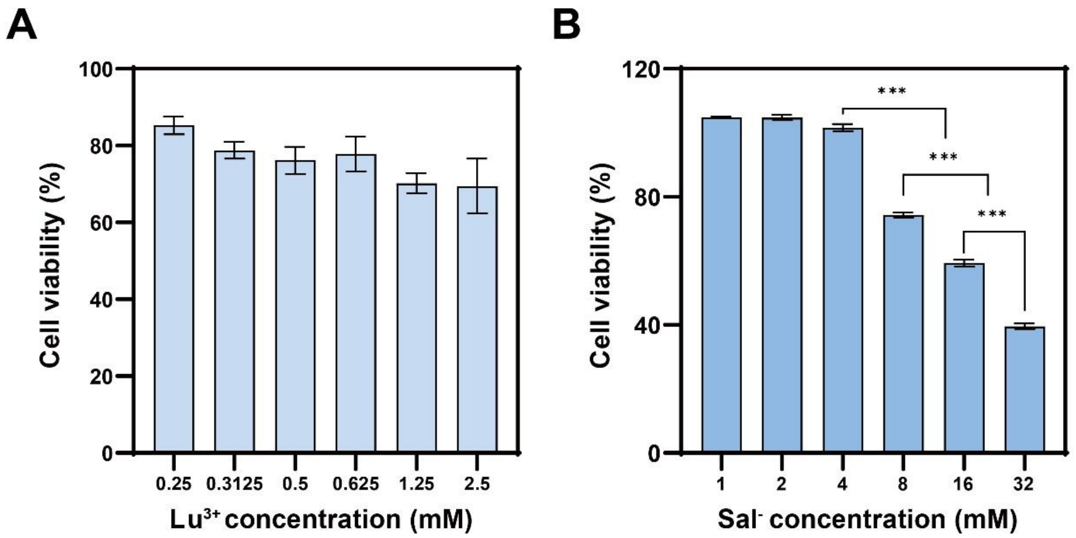

Figure S10. Cytotoxicity of 4T1 cells treated with (**A**) Lu^{3+} (0.25–2.5 mM) and (**B**) Sal^- (1–32 mM) ($n = 3$). Data are presented as mean ± SD; $***P < 0.001$.

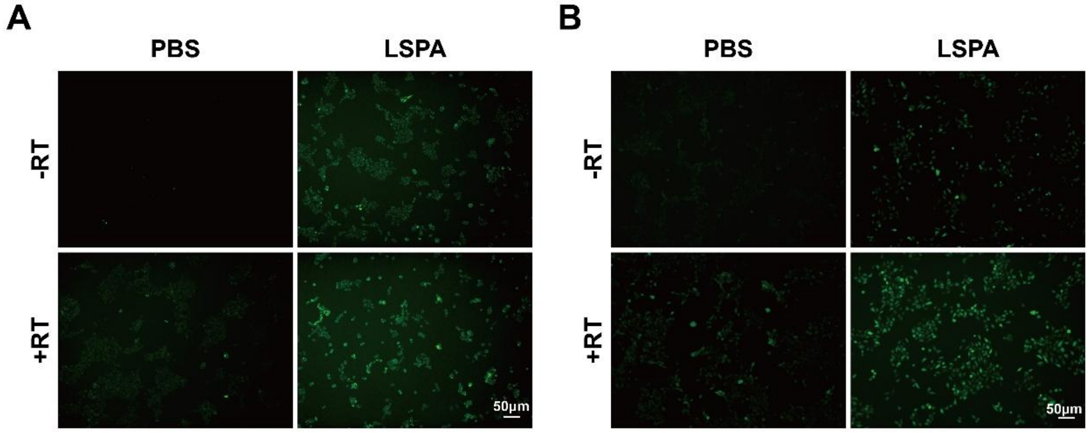

Figure S11. Confocal laser scanning microscopy (CLSM) images of 4T1 cells stained with **(A)** BBoxiProbe O22 probe (for $^1O_2$ detection) and **(B)** BBoxiProbe O27 probe (for •OH detection) after treatment with PBS (control) or LSPA, with or without 6 Gy X-ray irradiation (RT). Scale bar: 50 μm.

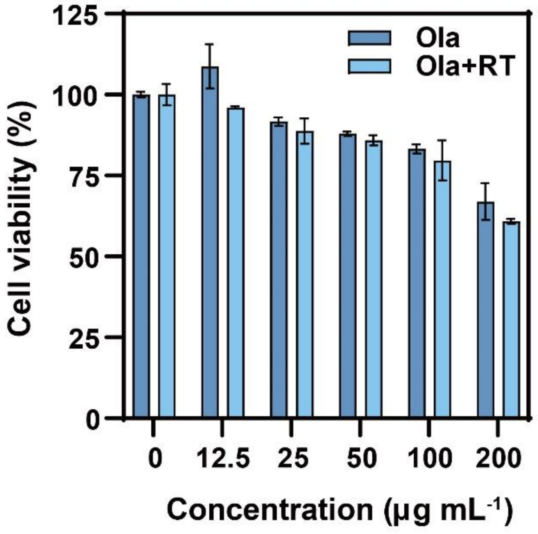

Figure S12. Cytotoxicity of 4T1 cells treated with increasing concentrations of Olaparib (Ola), with or without 6 Gy X-ray irradiation (RT, n = 3). Data are presented as mean ± SD.

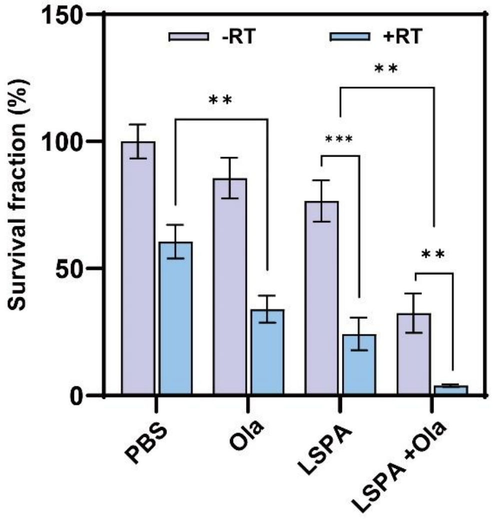

Figure S1. Clonogenic survival fraction (%) of 4T1 cells following the indicated treatments (n = 3). RT: 6 Gy X-ray irradiation; Ola: Olaparib. Data are presented as mean ± SD; $^{**}P < 0.01$; $^{***}P < 0.001$.

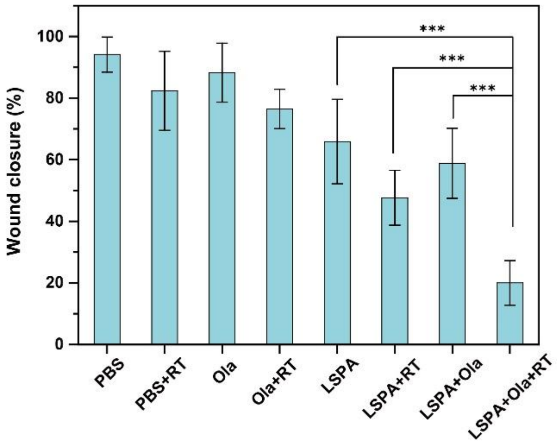

Figure S2. Percentages of wound closure in 4T1 cells following the indicated treatments ($n = 3$). RT: 6 Gy X-ray irradiation; Ola: Olaparib. Data are presented as mean ± SD; ***$P < 0.001$.

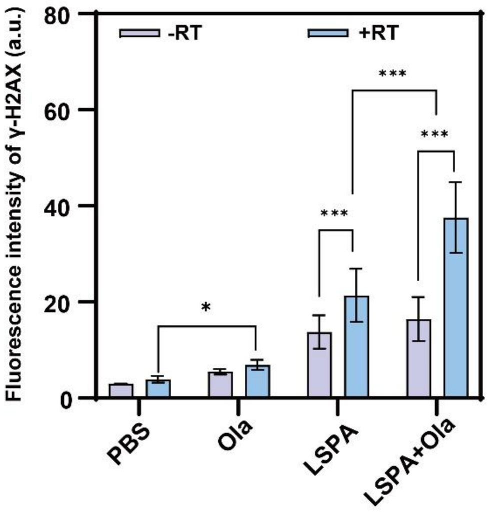

Figure S3. γ-H2AX staining fluorescence intensity in 4T1 cells following the indicated treatments ($n = 3$, fluorescence intensity per nucleus). RT: 6 Gy X-ray irradiation; Ola: Olaparib. Data are presented as mean ± SD; *$P < 0.05$; ***$P < 0.001$.

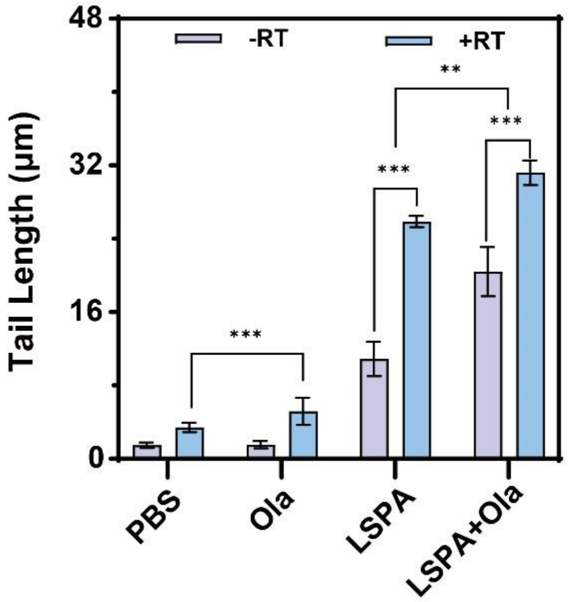

Figure S4. Quantification of DNA damage by comet tail length in 4T1 cells following the indicated treatments (n = 3). RT: 6 Gy X-ray irradiation; Ola: Olaparib. Data are presented as mean ± SD; **P < 0.01; ***P < 0.001.

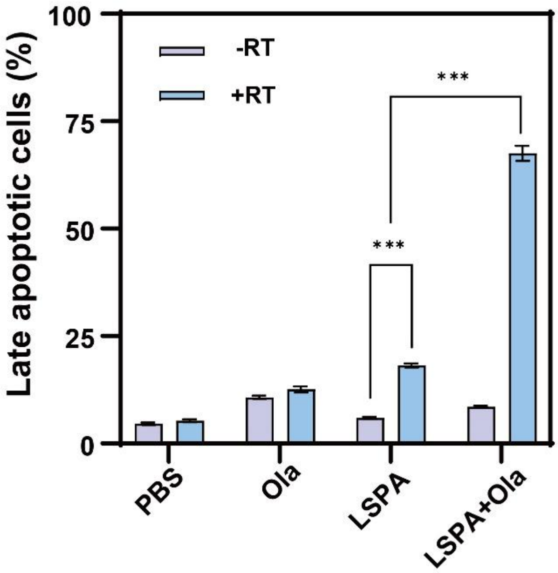

Figure S5. Late apoptosis (Annexin V^+ PI^+) quantification in 4T1 cells following the indicated treatments (n = 3). RT: 6 Gy X-ray irradiation; Ola: Olaparib. Data are presented as mean ± SD; ***P < 0.001.

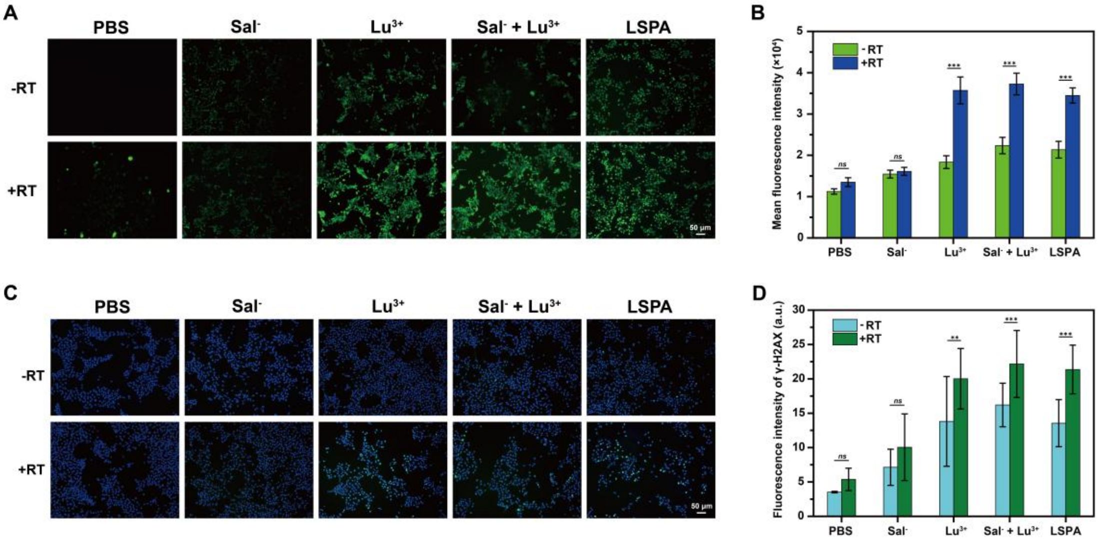

Figure S18. Verification of the *in vitro* radiosensitization effect of LSPA nanoparticles. **(A)** CLSM images of DCFH-DA-stained 4T1 cells (ROS imaging) following the indicated treatments with or without RT. Scale bar: 50 μm. **(B)** Corresponding fluorescence intensities of DCFH-DA-stained 4T1 cells treated with various treatments with or without RT ($n = 3$). **(C)** γ-H2AX immunofluorescence staining (a marker for DSBs) of 4T1 cells following the indicated treatments (DAPI counterstaining). Scale bar: 50 μm. **(D)** Corresponding γ-H2AX staining fluorescence intensities in 4T1 cells following the indicated treatments ($n = 3$, fluorescence intensity per nucleus). RT: 6 Gy X-ray irradiation. Data are presented as mean ± SD; *ns*: no significance; $**P < 0.01$; $***P < 0.001$.

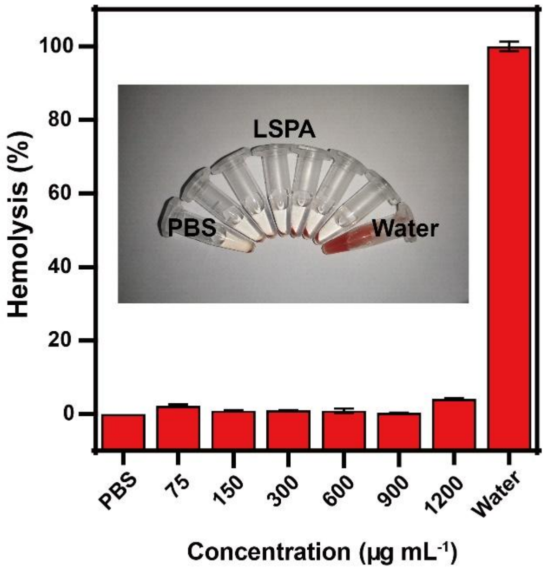

Figure S6. Hemocompatibility assessment of LSPA. Hemolysis rates (%) of red blood cells (RBCs) incubated with LSPA (75–1200 μg mL^{-1}) at 4 °C for 3 h ($n = 3$). Data are presented as mean ± SD.

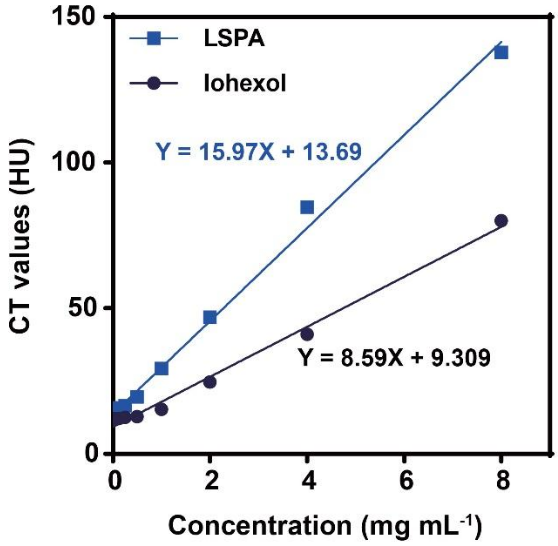

Figure S20. Contrast performance of LSPA versus Iohexol. CT signal enhancement as a function of concentration for LSPA and Iohexol using monoenergetic image reconstruction at 100 keV.

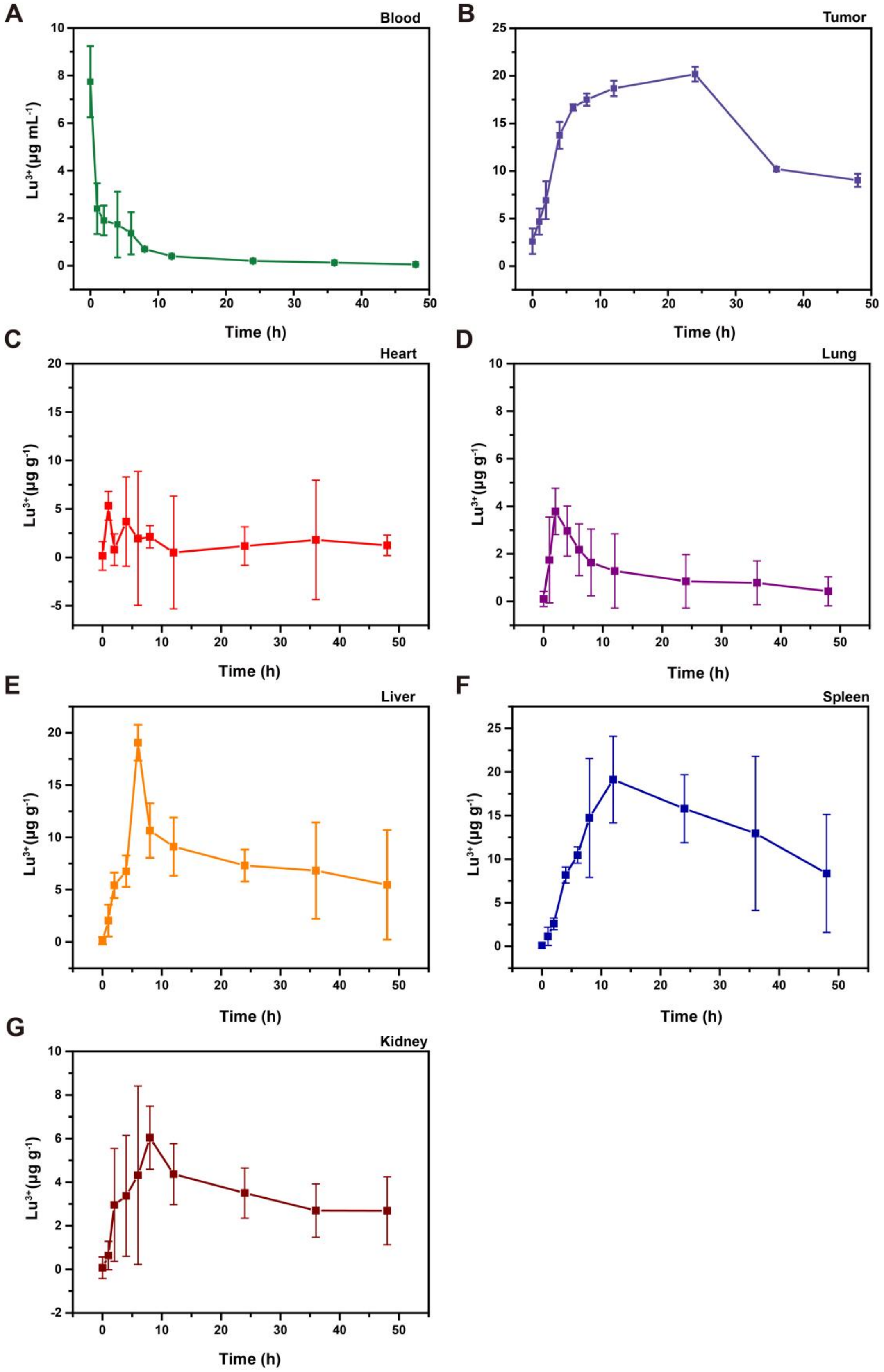

Figure S21. Time-dependent in vivo biodistribution of LSPA nanoparticles at 48 h post-injection. **(A)** blood; **(B)** tumor tissue; **(C)** heart; **(D)** lung; **(E)** liver; **(F)** spleen; and **(G)** kidney. Lu^{3+} concentrations were determined by ICP-MS (n = 3). Data are presented as mean ± SD.

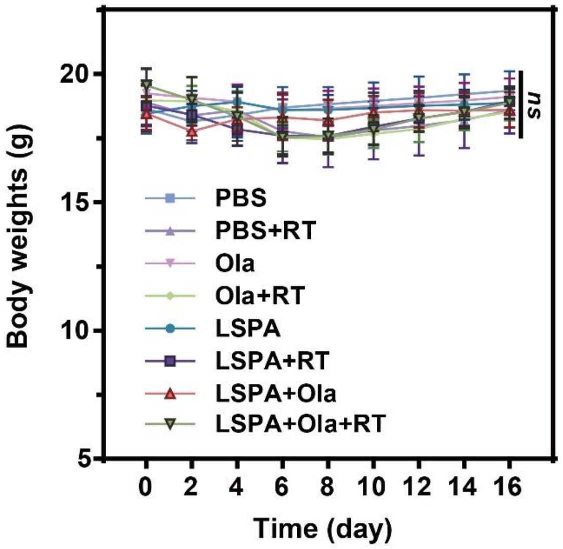

Figure S22. Body weight changes in 4T1 tumor-bearing mice over a 16-day period post-treatment (n = 5). RT: 6 Gy X-ray irradiation; Ola: Olaparib. Data are presented as mean ± SD; *ns*: no significance.

Table S1. Tumor growth inhibition (TGI) values across treatment groups (n = 5).

Treatment Groups	Primary tumor (%)		Distant tumor (%)	
	Mean	SD	Mean	SD
PBS	0	6.20	0	6.89
PBS + RT	45.42	2.76	45.60	2.24
Olaparib (Ola)	-0.45*	3.00	5.85	9.19
Ola + RT	56.32	2.19	56.34	2.00
LSPA	61.28	2.80	60.75	1.46
LSPA+RT	81.60	0.60	81.25	1.16
LSPA + Ola	76.00	1.68	82.25	0.58
LSPA + Ola + RT	89.70	1.52	91.99	0.38

* The negative TGI value observed in the Olaparib-alone group was not statistically significant compared to the control group ($P > 0.5$) and is attributed to normal inter-animal variation. RT: 6 Gy X-ray irradiation; Ola: Olaparib.

Table S2. Comparative analysis of nano-radiosensitizers and PARP inhibitor-enhanced radiotherapy studies.

**Therapeutic Strategy**	**Nanosensitizer / Drug**	**Radiation Dose**	**Key Mechanism**	**Primary Outcome**	**Reference**
LSPA + Olaparib + RT	LSPA (Lu-based)	6 Gy (fractionated)	ROS amplification; PARP inhibition; Synergistic cGAS-STING activation	Potent primary & abscopal tumor regression; Durable immune memory	This Work
Nano-radiosensitizer (classical high-Z) + RT	Gold nanoparticles (AuNP)	30 Gy (single dose)	High-Z photoelectric effect; local dose enhancement	Improved tumor control/survival vs RT alone	The use of gold nanoparticles to enhance radiotherapy in mice (DOI: 10.1088/0031-9155/49/18/N03)
Self-targeting Nano-prodrug + RT	Platinum(IV) amphiphilic prodrug nano-assembly	6 Gy (single dose)	Radiosensitization (from Platinum); Synergistic chemotherapy release	Synergistic and safe chemoradiotherapy for hepatocellular carcinoma	Self-targeting platinum(IV) amphiphilic prodrug nano-assembly as radiosensitizer for synergistic and safe chemoradiotherapy of hepatocellular carcinoma (DOI: 10.1016/j.biomaterials.2022.121793)
Nano-radiosensitizer (Gd, clinical-stage) + RT	AGuIX (ultrasmall Gd-based nanoparticle)	Clinical WBRT (30 Gy in 10 fractions, brain metastases)	High-Z radiosensitization; MRI-visible; rapid renal clearance	Feasibility/safety; preliminary efficacy in brain metastases	Theranostic AGuIX nanoparticles as radiosensitizer: A phase I, dose- escalation study in patients with multiple brain metastases (NANO-RAD trial) (DOI: 10.1016/j.radonc.2021.04.021)
Nano-radiosensitizer (HfO_2, clinical-stage) + RT	NBTXR3 (HfO_2 nanoparticle; intratumoral)	50 Gy in 25 fractions (pre-operative RT, soft-tissue sarcoma)	High-Z dose enhancement (secondary electron emission)	Higher pathological response vs RT alone; acceptable safety	First-in-human study testing a new radioenhancer using nanoparticles (NBTXR3) activated by radiation therapy in patients with locally advanced soft tissue sarcomas (DOI: 10.1158/1078-0432.CCR-16-1297)

Nano-radiosensitizer (Hf-porphyrin nMOF) + RT + ICB	Hf-based nMOF	8 Gy × 3 (typical preclinical regimen)	High-Z radiosensitization + radiodynamic ROS; ICD/STING activation	Enhanced local control and abscopal effect with anti-PD-L1	Nanoscale metal-organic frameworks enhance radiotherapy to potentiate checkpoint blockade immunotherapy (DOI: 10.1038/s41467-018-04703-w)
PARP Inhibitor + RT (General)	Olaparib	8 Gy × 3 or single 12 Gy (preclinical)	Inhibition of DNA single-strand break repair; cGAS-STING activation	Potent systemic antitumor immunity and abscopal effects	Olaparib enhances radiation-induced systemic anti-tumor effects via activating STING-chemokine signaling in hepatocellular carcinoma (DOI: 10.1016/j.canlet.2023.216507)

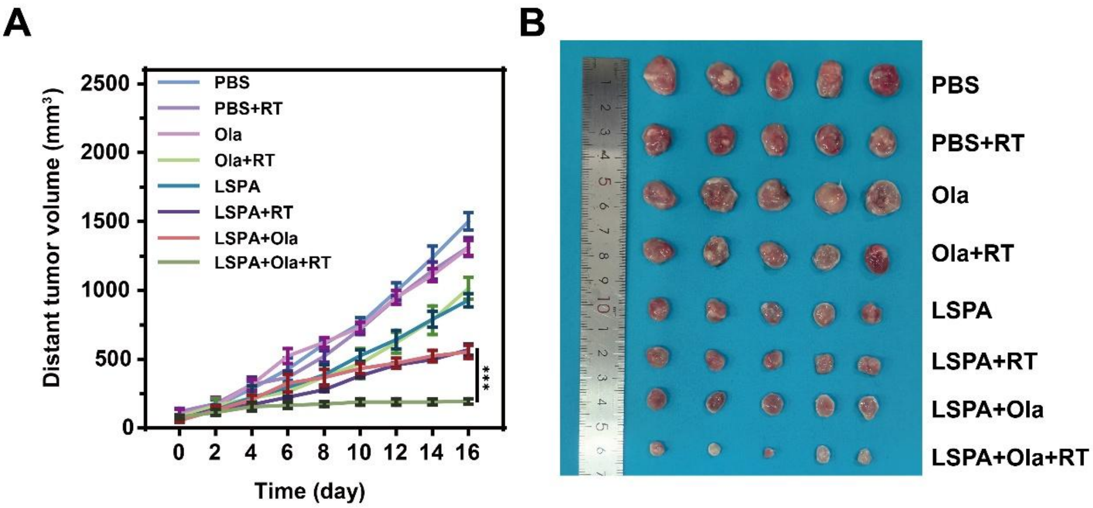

Figure S23. (A) Growth curves of distant tumors in 4T1 tumor-bearing mice over a 16-day treatment period (*n* = 5). **(B)** Representative photographs of excised distant tumors. RT: 6 Gy X-ray irradiation; Ola: Olaparib. Data are presented as mean ± SD; ****P* < 0.001.

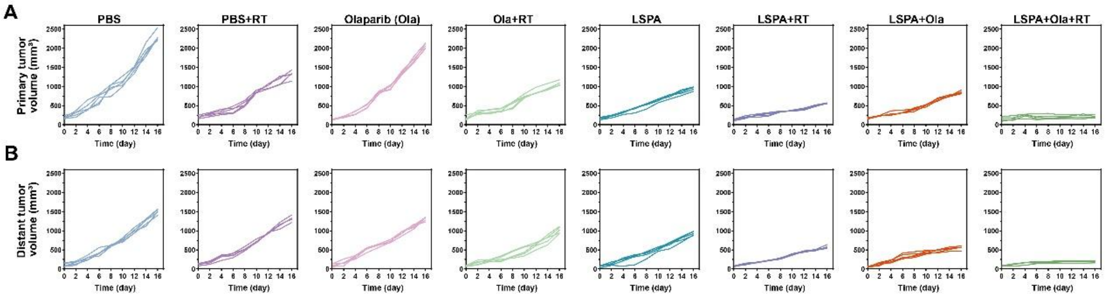

Figure S24. Individual tumor growth kinetics. **(A)** Primary and **(B)** distant tumor growth curves in 4T1 tumor-bearing mice during the 16-day treatment (n = 5). RT: 6 Gy X-ray irradiation; Ola: Olaparib. Data are presented as mean ± SD.

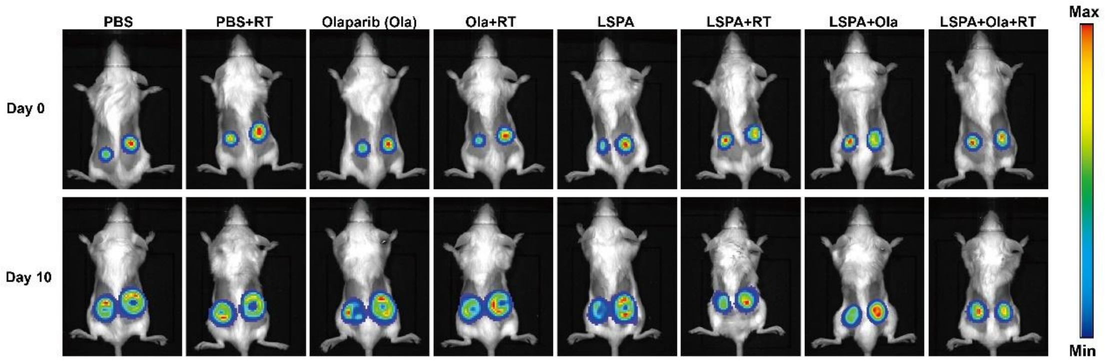

Figure S25. Representative *in vivo* bioluminescence images show primary (right) and distant (left) tumors in 4T1-Luc tumor-bearing mice following the indicated treatments. RT: 6 Gy X-ray irradiation; Ola: Olaparib.

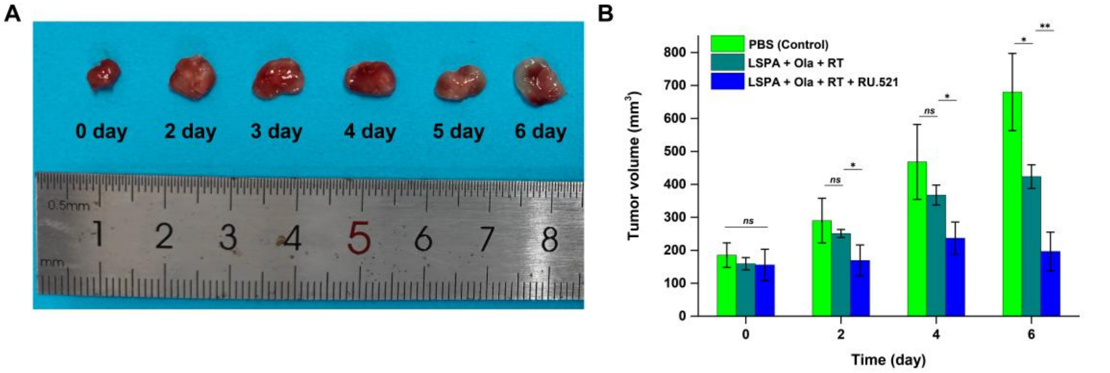

Figure S26. Verification of the therapeutic mechanism underlying cGAS-STING pathway activation. **(A)** Representative photograph of excised tumors following treatment within the first 6 days. **(B)** Corresponding tumor volumes following the indicated treatments during the first 6 days.

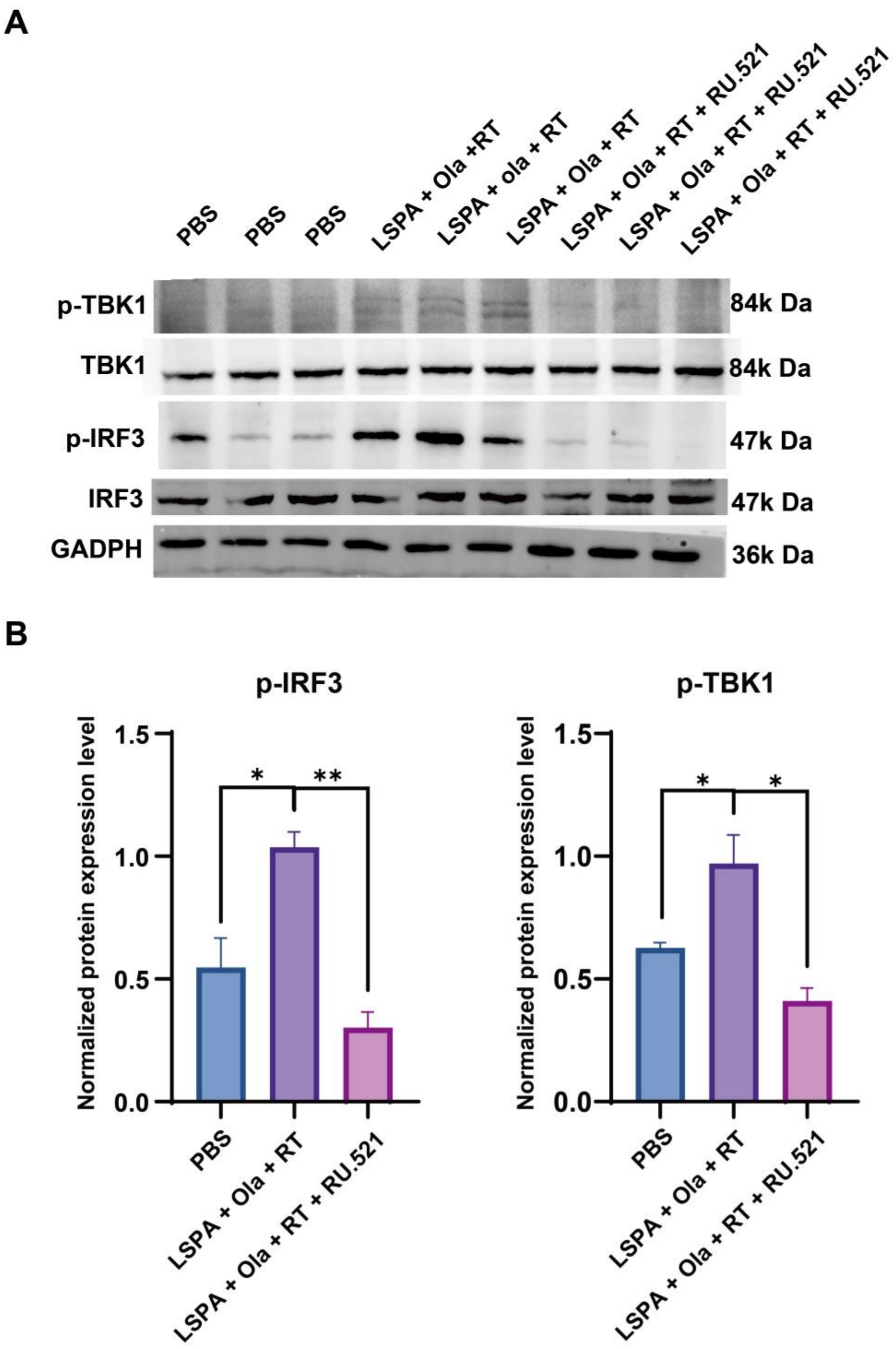

Figure S27. Western Blot for STING Pathway Validation. **(A)** Western blot analysis of cGAS-STING pathway biomarkers (p-IRF3, p-TBK1) in tumor lysates following the indicated treatments. (B) Quantification of p-TBK1 and p-IRF3 biomarkers levels (n = 3). Ola: Olaparib; RT: 6 Gy X-ray irradiation. Data are presented as mean ± SD. *$P < 0.05$; **$P < 0.01$.

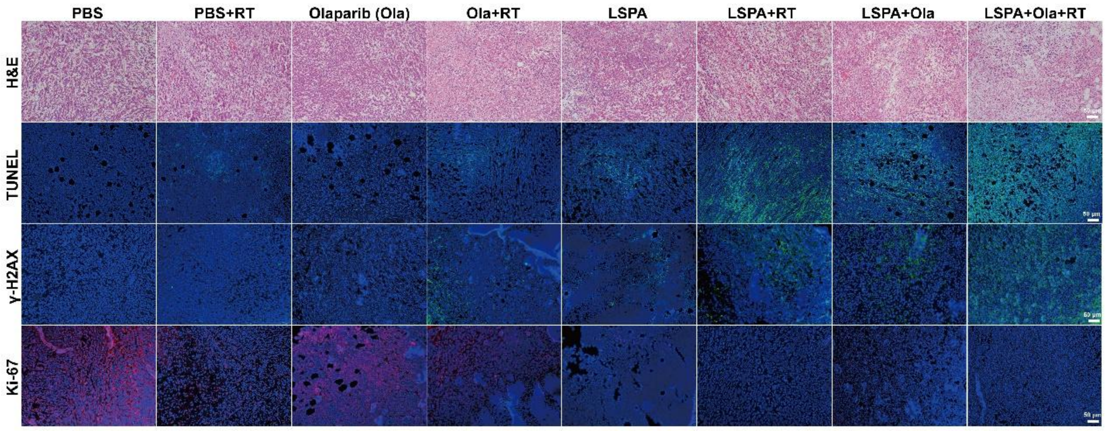

Figure S28. Immunohistochemical analysis of distant tumors reveals treatment-induced effects (H&E, TUNEL, γ-H2AX, and Ki-67 staining) following the indicated treatments. RT: 6 Gy X-ray irradiation; Ola: Olaparib. Scale bar: 50 μm.

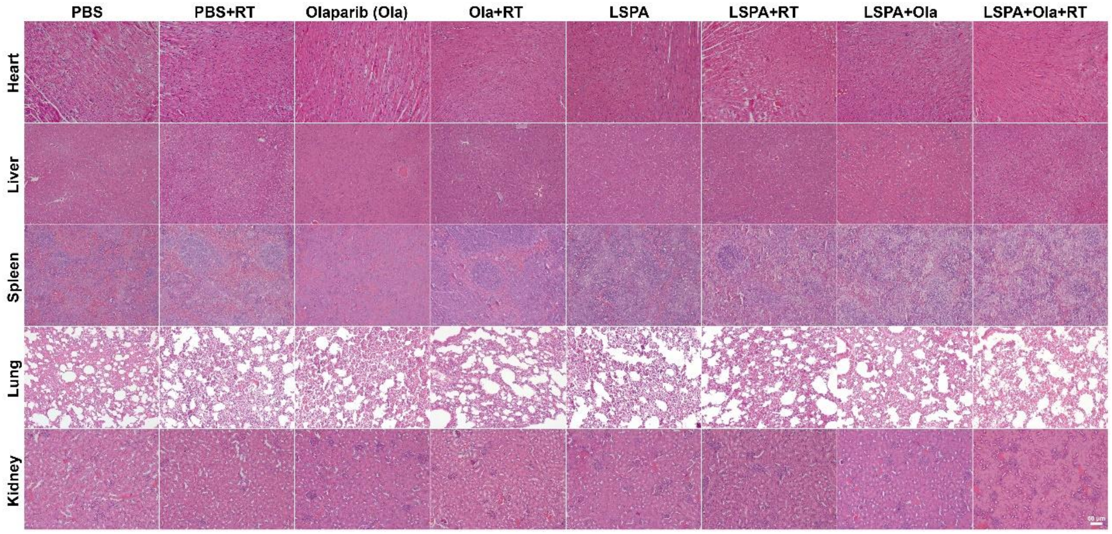

Figure S29. Histopathological evaluation (H&E staining) of major organs (heart, liver, spleen, lung, and kidney) from 4T1 tumor-bearing mice following the indicated treatments. RT: 6 Gy X-ray irradiation; Ola: Olaparib. Scale bar: 50 μm.

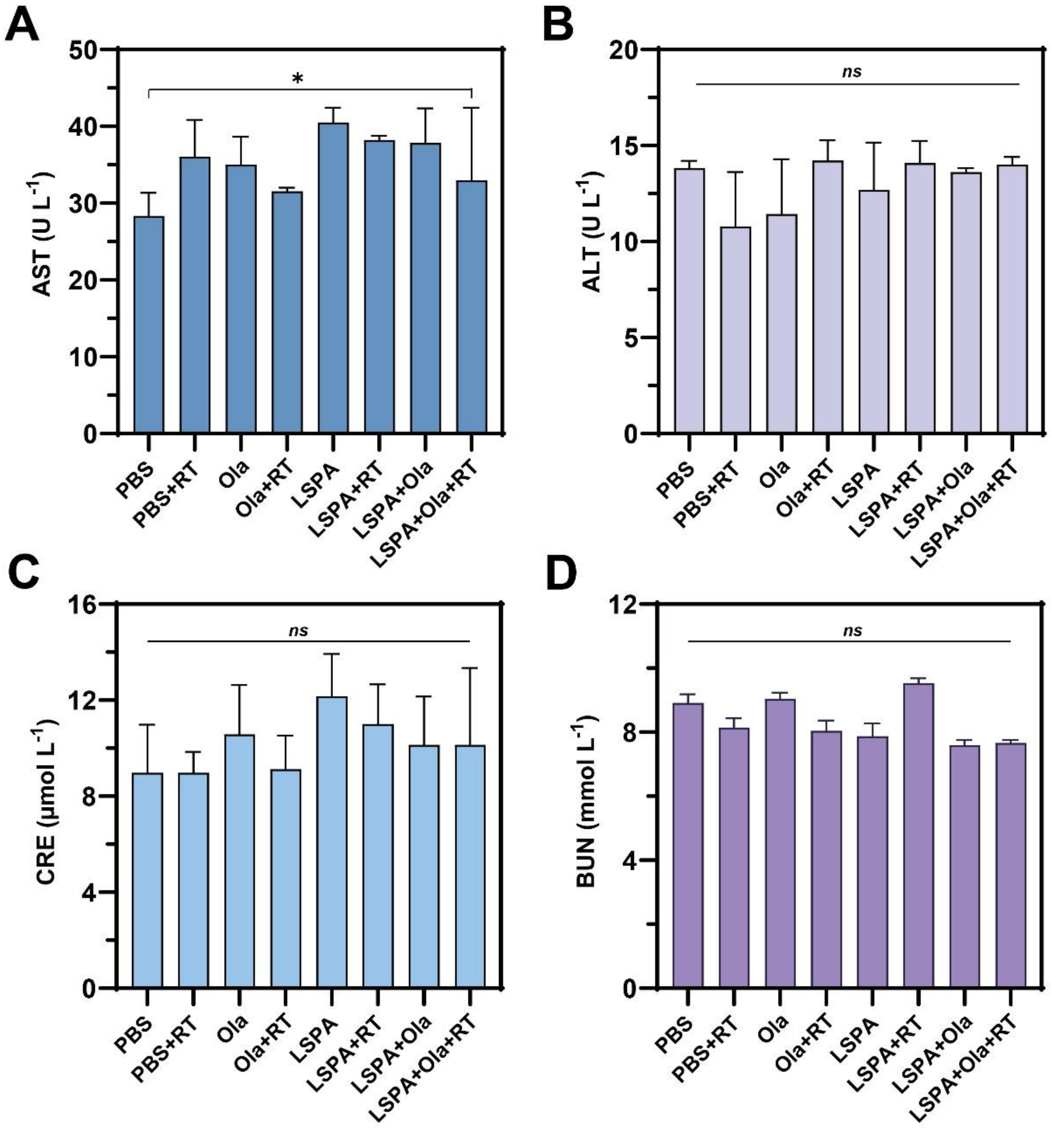

Figure S30. Serum biochemical analyses of 4T1 tumor-bearing mice after 16 days of the indicated treatments. **(A-B)** Liver function markers: aspartate aminotransferase (AST, n = 3) and alanine aminotransferase (ALT, n = 3). **(C-D)** Kidney function markers: creatinine (CRE, n = 3) and blood urea nitrogen (BUN, n = 3). RT: 6 Gy X-ray irradiation; Ola: Olaparib. Data are presented as mean ± SD; *ns*: no significance. *$P < 0.05$.

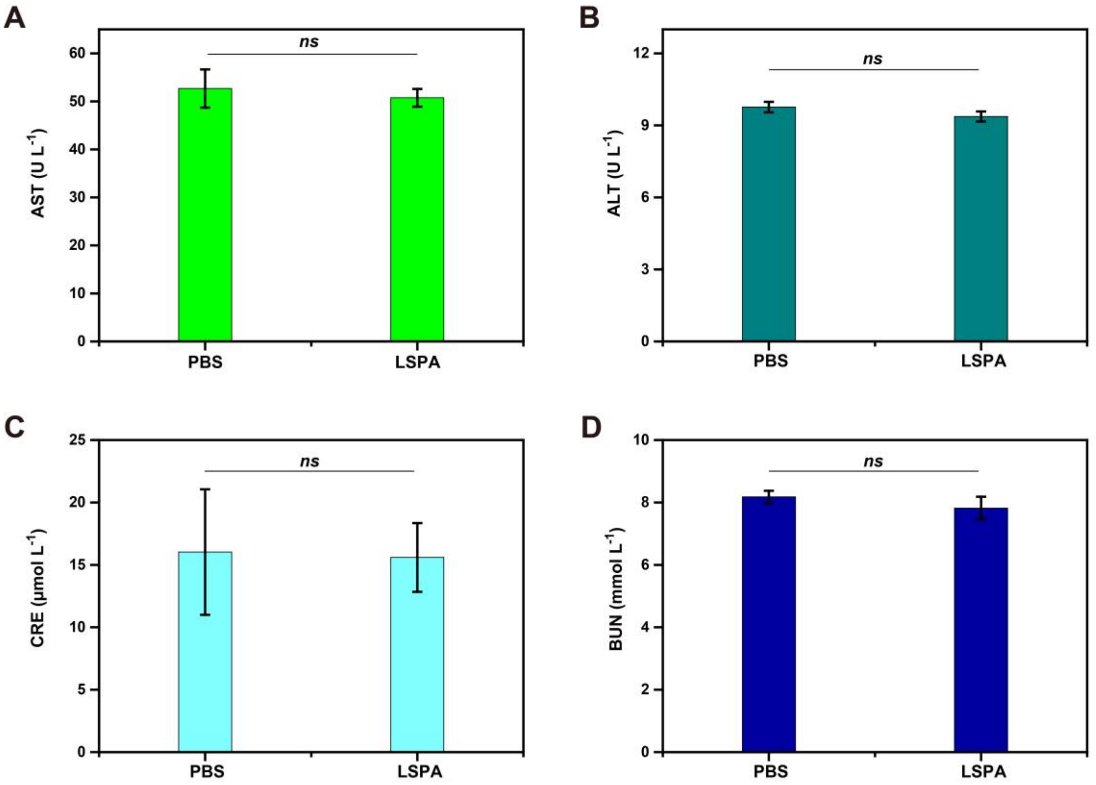

Figure S31. Long-term *in vivo* toxicity assessment of LSPA nanoparticles. Serum biochemistry analysis of healthy BALB/c mice after intravenous injection of LSPA nanoparticles or PBS every 7 days for 3 doses over a 28-day period. The levels of **(A)** aspartate aminotransferase (AST), (B) alanine aminotransferase (ALT), (C) creatinine (CRE), and (D) blood urea nitrogen (BUN) were measured. Data are presented as mean ± SD (n = 3); *ns*: no significance.

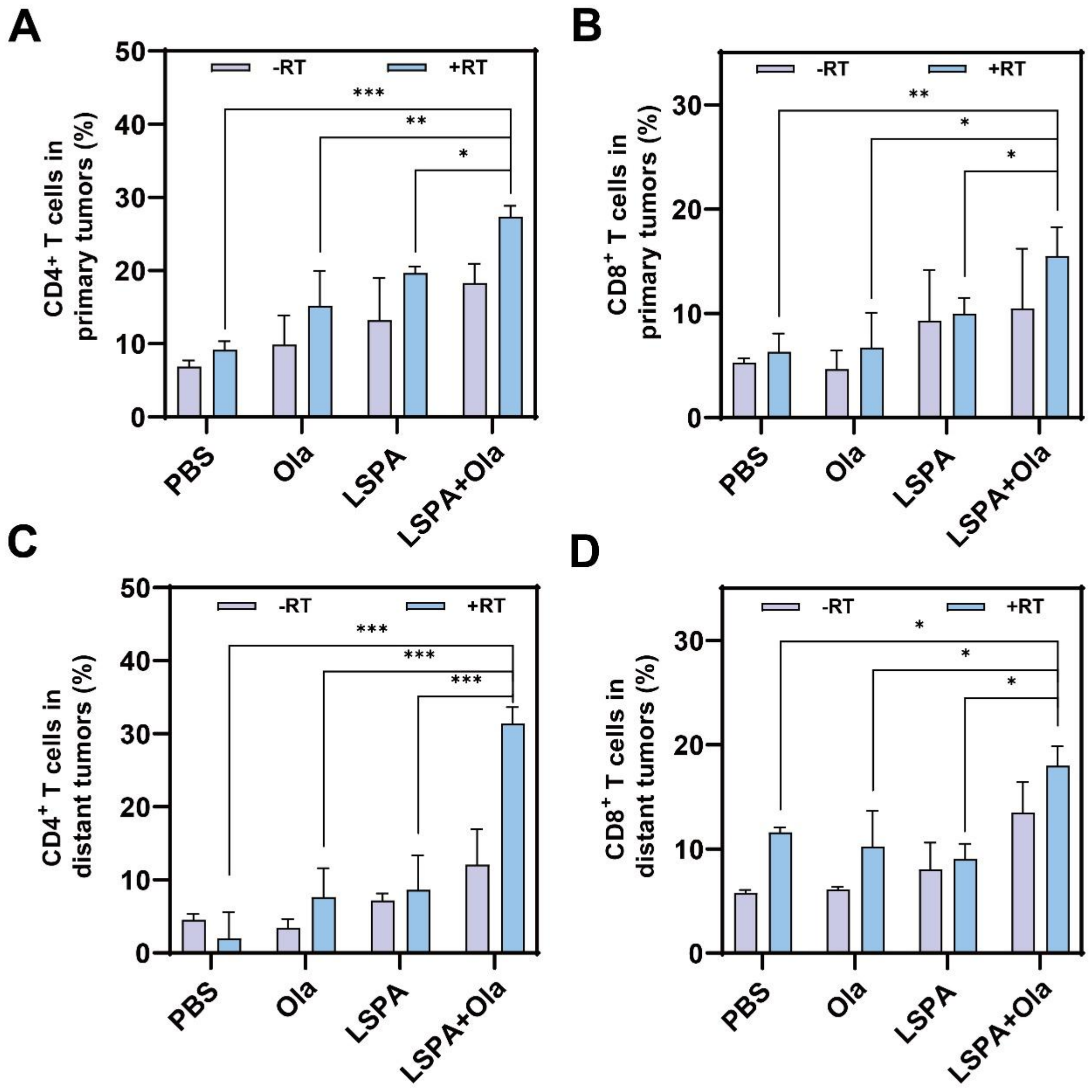

Figure S32. Immune cell infiltration analysis in tumor models. **(A)** $CD4^+$ T cell infiltration in primary tumors (n = 3). **(B)** $CD8^+$ T cell infiltration in primary tumors (n = 3). **(C)** $CD4^+$ T cell infiltration in distant tumors (n = 3). **(D)** $CD8^+$ T cell infiltration in distant tumors (n = 3). RT: 6 Gy X-ray irradiation; Ola: Olaparib. Data are presented as mean ± SD, *$P < 0.05$; **$P < 0.01$; ***$P < 0.001$.

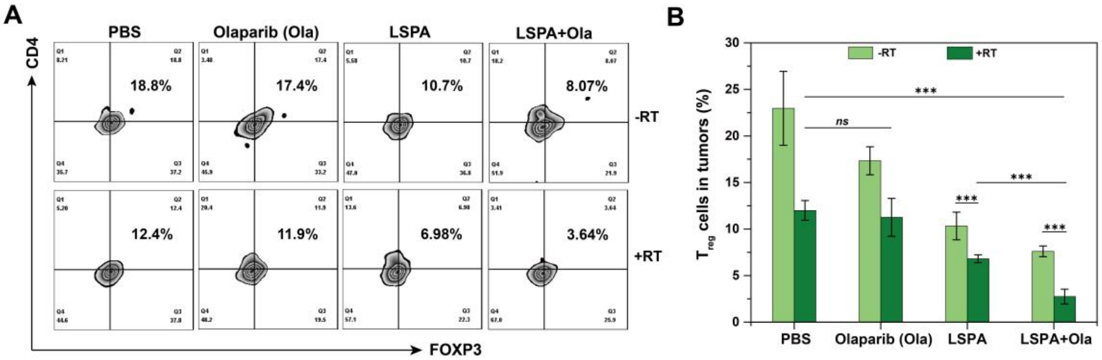

Figure S33. Flow cytometric analysis of tumor-infiltrating regulatory T (T_{reg}) cells. **(A)** Representative flow cytometry plots of T_{reg} cells ($CD4^{+}$ $FOXP3^{+}$). **(B)** Quantification of tumor-infiltrating T_{reg} cells (n = 3). RT: 6 Gy X-ray irradiation; Ola: Olaparib. Data are presented as mean ± SD; *ns*: no significance; ****P* < 0.001.

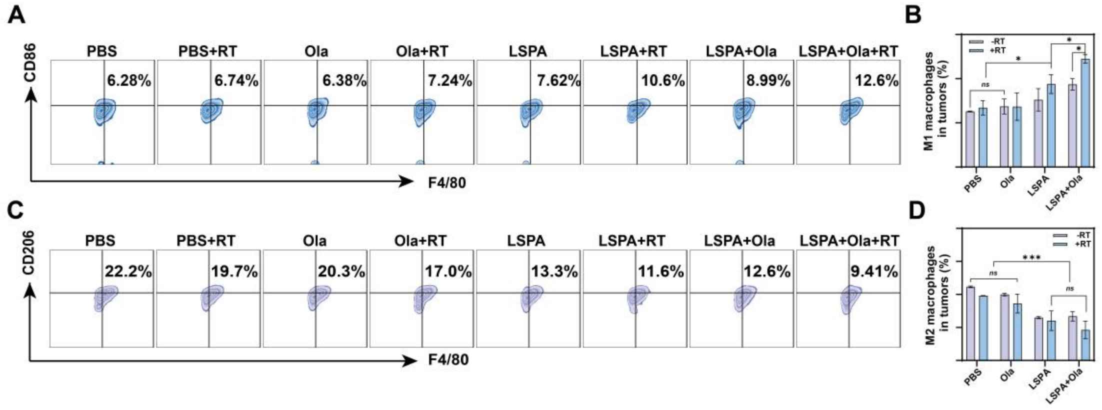

Figure S34. Polarization analysis of tumor-associated macrophages (TAMs). **(A)** Representative flow cytometry plots of M1-polarized TAMs ($F4/80^{+}$ $CD86^{+}$). **(B)** Quantification of M1-polarized TAMs in tumors (n = 3). **(C)** Representative flow cytometry plots of M2-polarized TAMs ($F4/80^{+}$ $CD206^{+}$). **(D)** Quantification of M2-polarized TAMs in tumors (n = 3). RT: 6 Gy X-ray irradiation; Ola: Olaparib. Data are presented as mean ± SD; *ns*: no significance; **P* < 0.05, ****P* < 0.001.

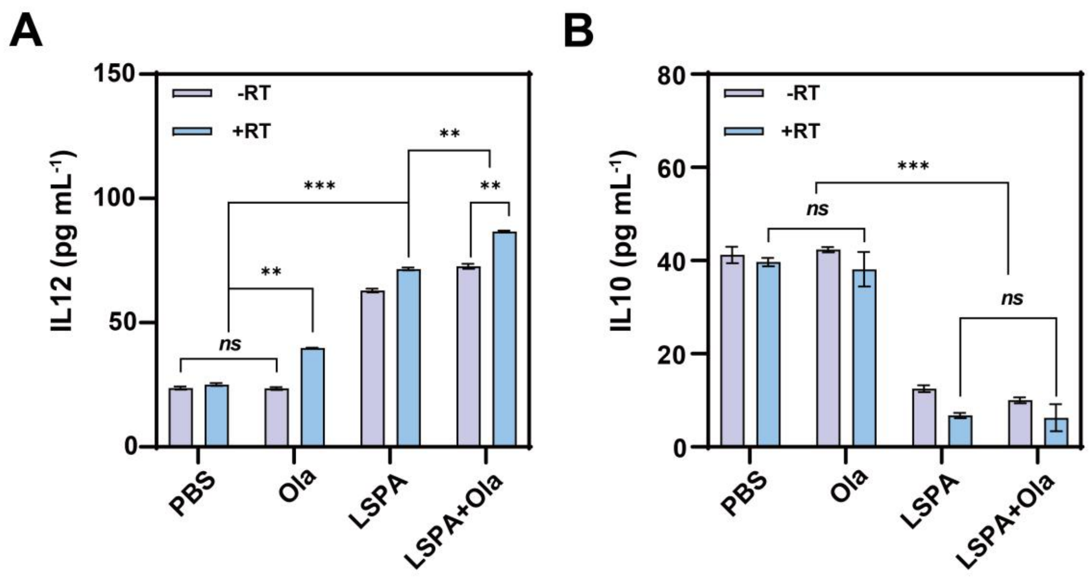

Figure S35. Serum cytokine profiling in 4T1 tumor-bearing mice. Serum levels of **(A)** IL-12 (pro-inflammatory) and **(B)** IL-10 (anti-inflammatory) were quantified by ELISA following the indicated treatments ($n = 3$). RT: 6 Gy X-ray irradiation; Ola: Olaparib. Data are presented as mean ± SD; *ns*: no significance; $^{**}P < 0.01$; $^{***}P < 0.001$.

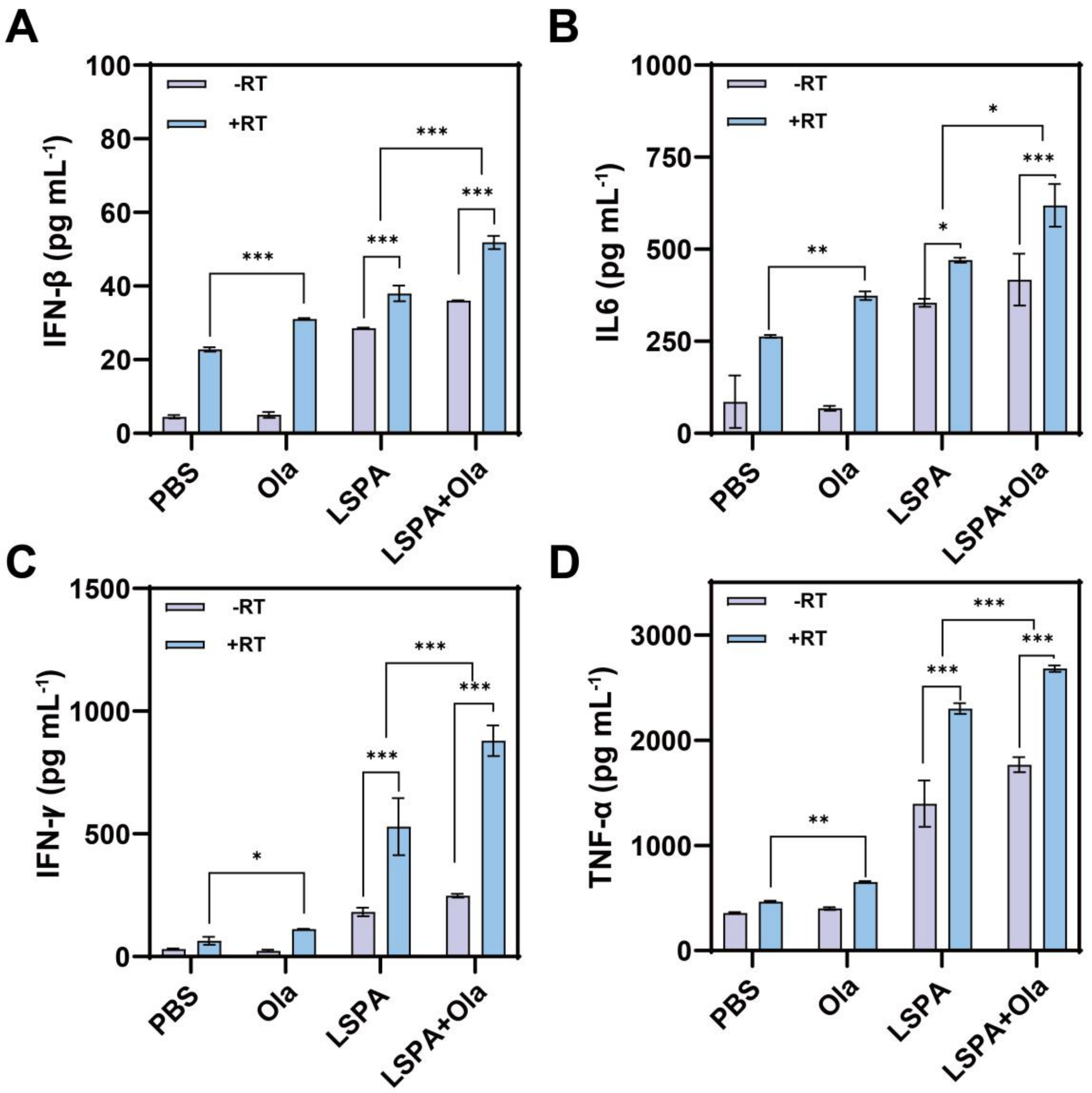

Figure S36. Serum cytokine analysis in 4T1 tumor-bearing mice. Quantification of **(A)** IFN-β (interferon-β), **(B)** IL-6 (interleukin-6), **(C)** IFN-γ (interferon-γ), and **(D)** TNF-α (tumor necrosis factor-α) by ELISA following the indicated treatments (n = 3). RT: 6 Gy X-ray irradiation; Ola: Olaparib. Data are presented as mean ± SD. *$P < 0.05$; **$P < 0.01$; ***$P < 0.001$.
